# Supplementary material for: Designing multi-metal-site nanosheet catalysts for CO2 photoreduction to ethylene
Source: Nat Commun. 2025 Jul 15;16:6500. doi: 10.1038/s41467-025-61850-7 (PMC12259931; doi:10.1038/s41467-025-61850-7)
Supplement: Supplementary file 1 — Supplementary Information [file 41467_2025_61850_MOESM1_ESM.pdf]

Supplementary Information

Designing Multi-Metal-Site Nanosheet Catalysts for CO_2 Photoreduction to Ethylene

Xiaodong Li[1†], Li Li[2†], Xiaohui Liu[3†], Jiaqi Xu[4, 5†], Xingyuan Chu[3], Guangbo Chen[3], Dongqi Li[3], Mingchao Wang[3], Xia Wang[6], Chandrasekhar Naisa[1], Jing Gao[4], Yongfu Sun[2*], Michael Grätzel[4*] and Xinliang Feng[1, 3*]

[1]Max Planck Institute of Microstructure Physics, Weinberg 2, Halle 06120, Germany.

[2]Hefei National Research Center for Physical Sciences at Microscale, University of Science and Technology of China, 230026, P. R. China.

[3]Faculty of Chemistry and Food Chemistry & Center for Advancing Electronics Dresden (cfaed), Dresden University of Technology, Dresden 01062, Germany.

[4]Laboratory of Photonics and Interfaces, École Polytechnique Fédérale de Lausanne, 1015 Lausanne, Switzerland.

[5]Key Laboratory of Green Chemistry & Technology of Ministry of Education, College of Chemistry, Sichuan University, Chengdu, Sichuan 610064, P. R. China.

[6]Max Planck Institute for Chemical Physics of Solids, Dresden 01187, Germany.

[†]These authors contributed equally: Xiaodong Li, Li Li, Xiaohui Liu, Jiaqi Xu.

*Corresponding author. Email: yfsun@ustc.edu.cn, michael.graetzel@epfl.ch, Xinliang.Feng@tu-dresden.de

Supplementary Notes

Supplementary Note 1: For DFT calculations of $Cu_2M_iM_jS_4$ (M_i=Mn, Fe, Co, Ni, Zn; M_j=Ge, Sn) models, all parameter settings are the same as those used for Cu_2ZnSnS_4 models. The crystal lattice parameters of $Cu_2M_iM_jS_4$ bulk are as follows: a = b = 5.5651 Å, c = 10.9141 Å ($\alpha = \beta = \gamma = 90^{\circ}$) for Cu_2MnSnS_4 (space group (I-42m (121))); a = b = 5.4727 Å, c = 10.9290 Å ($\alpha = \beta = \gamma = 90^{\circ}$) for Cu_2FeSnS_4 (space group (I-42m (121))); a = b = 5.5651 Å, c = 10.9141 Å ($\alpha = \beta = \gamma = 90^{\circ}$) for Cu_2CoSnS_4 (space group (I-42m (121))); a = b = c = 5.4334 Å ($\alpha = \beta = \gamma = 90^{\circ}$) for Cu_2NiSnS_4 (space group (F-43m (216))); a = b = 5.3171 Å, c = 10.8103 Å ($\alpha = \beta = \gamma = 90^{\circ}$) for Cu_2ZnGeS_4 (space group (I-42m (121))). The theoretical approach is based on the GGA with on-site Coulomb interaction parameter (GGA + U method), in which an effective U-J parameter of 5.2, 4.0, 4.0, 6.7, 5.5, 6.5, 3.5 eV was applied to improve the description the 3d states of Cu, Mn, Fe, Co, Ni, Zn and Sn, respectively.[1, 2, 3, 4, 5, 6, 7] For Cu_2NiZnS_4 slab, the exposed surface along (111) direction was first built and converted into the similar slab structure with the Cu_2ZnSnS_4 (112) model by matrix transformation.

Supplementary Note 2: we first took the CZTS as an example and constructed its corresponding bulk configuration, as shown in Supplementary Figure 1a. From the calculated DOS (Supplementary Figure 1b), we determined its band gap to be approximately 1.46 eV, which is consistent with the previous experimental value.[8] Subsequently, we evaluated the surface energies for the most common planes with different exposed atoms, including (001), (100) and (112).[9, 10] As illustrated in Supplementary Figure 2-4, we found that the (112) plane, with sulfur atoms exposed in the outermost layer (referred to as 112-S), exhibits the lowest surface energy of 0.719 J/m^2. These results indicate that the 112-S structure represents the most stable surface and offers the most theoretically feasible pathway for the preparation of the corresponding 2D configuration. Considering the propensity of surface S atoms in the 2D configuration to migrate and create vacancy defects,[11, 12] we then conducted calculations to determine the defect formation energy of S vacancy at different positions. As depicted in Supplementary Figure 5, the defective structure with an S vacancy in position 1 exhibits a negative formation energy of -0.37 eV, signifying the spontaneous formation of the S defect at this specific location. To further confirm the structural stability of this S-defect configuration, we carried out the molecular dynamics (MD) simulations at a temperature of 300 K. As shown in Supplementary Figure 6, the energy of the specific structure stabilizes at

approximately -210.08 eV, without any significant structural reconstruction or collapse. This result underscores the stability of the 2D S-defect CZTS (CZTS-S_v) slab at room temperature, affirming its suitability for experimental synthesis.

To elucidate the structural and electronic properties of $Cu_2M_iM_jS_4$-S_v slabs, we analyzed their charge density distribution, Bader charge, and bond lengths. As depicted in Supplementary Figure 7-12, the charge density of Cu atoms near the S defect within the $Cu_2M_iM_jS_4$-S_v slabs is notably higher. These electron-rich Cu sites in the $Cu_2M_iM_jS_4$-S_v slabs prove advantageous for facilitating intermediate adsorption and the C-C coupling process.[13] The Cu-Cu bond length near the S defect is also measured to be larger in the $Cu_2M_iM_jS_4$-S_v slabs, compared to that in the pristine structure. The elongated distance between active Cu atoms can minimize steric hindrance, favoring the formation of C-C* intermediates and further promoting the generation of C_{2+} products.[14]

To analyze the suitability of the $Cu_2M_iM_jS_4$-S_v nanosheets for CO_2 photoreduction, we took the CZTS slab as an example. The band gap and edge positions were further calculated, which play a pivotal role for light absorption, electron excitation and charge transport. By combining the DOS and the average electrostatic potential, we delineated the band structure of the CZTS and CZTS-S_v slab as displayed in Supplementary Figure 24. The conduction band maximum (CBM) of the CZTS-S_v slab surpasses the potential required for CO_2 reduction (e.g. 0.14 V for C_2H_4 generation), while the valence band minimum (VBM) falls below the potential for H_2O oxidation (e.g. 1.23 V for O_2 generation). This result suggests the capability of CZTS-S_v slab for simultaneous CO_2 reduction and H_2O oxidation. Notably, the emergence of I and II peaks within the band gap of CZTS-S_v slab signifies the presence of surface states and defect states, respectively. In contrast, when examining the band structure of the pristine CZTS slab for comparison, only surface states (I) are detected within the band gap.

Supplementary Note 3: Calculation of turnover frequency (TOF)[15, 16]:

$$\text{TOF} = \frac{number\ of\ produced\ molecules}{\text{number of active sites} * \text{total reaction time}} \quad \text{(S1)}$$

$$\text{Number of produced molecules per hour} = \text{ethylene rate} * N_A \quad \text{(S2)}$$

Number of active sites per milligram = active area from CO_2 adsorption isotherms ($m^2\ g^{-1}$) * density of

active sites (m^{-2}) (S3)

$$\text{Density of active sites} = \frac{1}{\text{area of simulated exposed facet}} \quad (S4)$$

Ethylene rate in 12h = 25.16 μmol g^{-1} h^{-1}

$N_A = 6.022 * 10^{17}$ μmol^{-1}

Active area from CO_2 adsorption isotherms = 33.1022 m^2 g^{-1}

Area of simulated exposed facet = 7.6860 Å * 13.3855 Å = 1.02881 * 10^{-18} m^2

So the TOF is calculated to 0.47 h^{-1}, while the corresponding turnover number (TON) is 5.64 (12h).

Supplementary Note 4: Cost analysis for synthesizing CZTS-S_v nanosheet:

Chemical	Price	Company (Part Number)
Copper(II) acetate monohydrate	0.1576 € /g	Sigma-Aldrich (1027109050-50KG)
Zinc acetate dihydrate	0.2044 € /g	Sigma-Aldrich (383058-2.5KG)
Tin(IV) chloride pentahydrate	0.217 € /g	Sigma-Aldrich (244678-1KG)
Thioacetamide	0.206 € /g	Sigma-Aldrich (172502-500G)
Ethylene glycol	51.8 € /L	Sigma-Aldrich (102466-5L)

The usage of reactants includes 91 mg copper acetate monohydrate, 55 mg zinc acetate dehydrate, 88 mg tin chloride pentahydrate, 80 mg thioacetamide and 40 mL ethylene glycol. The production of CZTS-S_v nanosheet is around 80 mg and the price is 26.66 € /g according to the reactant price. Compared with the precious metal, like Au (90.04 € /g), the price of obtained sample is more than 3 times lower, indicating the sustainability of our catalyst.

For scalable synthesis: we used 100 mL Tefon-lined autoclave to synthesize CZTS-S_v nanosheet with the double reactants and got around 160 mg sample with the same conversion rate, confirming its scalability of this method. Due to capacity limitations, we used multiple 50 and 100 mL Tefon-lined autoclave to synthesize 2 g of sample at a time (Supplementary Figure 31c).

Supplementary Figures

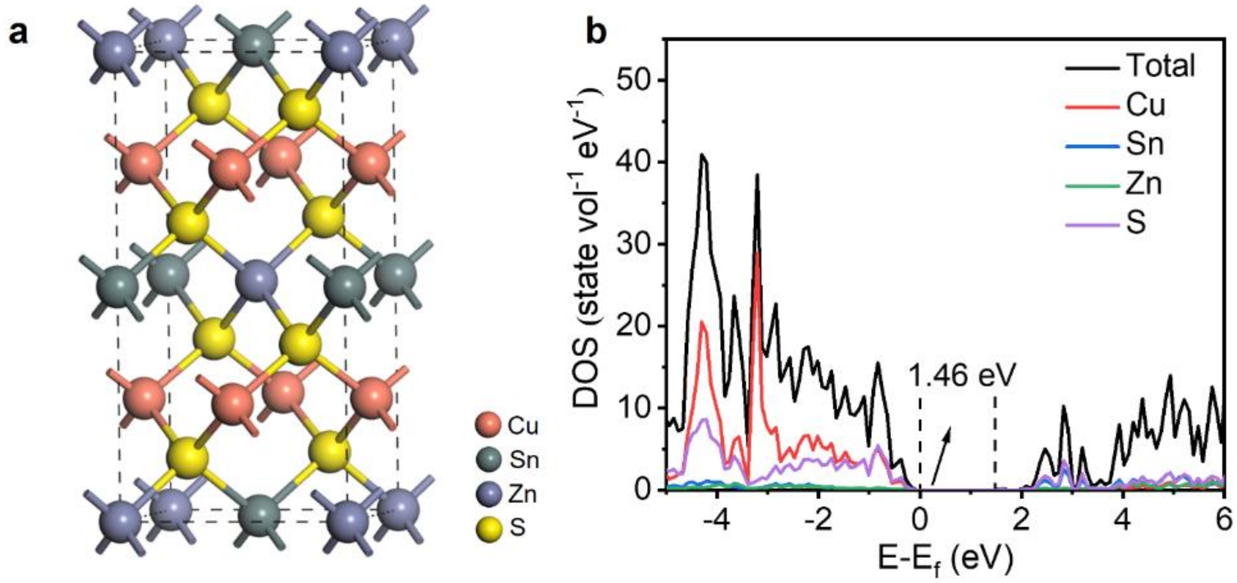

Supplementary Figure 1. Calculations for the Cu_2ZnSnS_4 (CZTS) bulk. (a) The theoretical model and (b) calculated density of states (DOS).

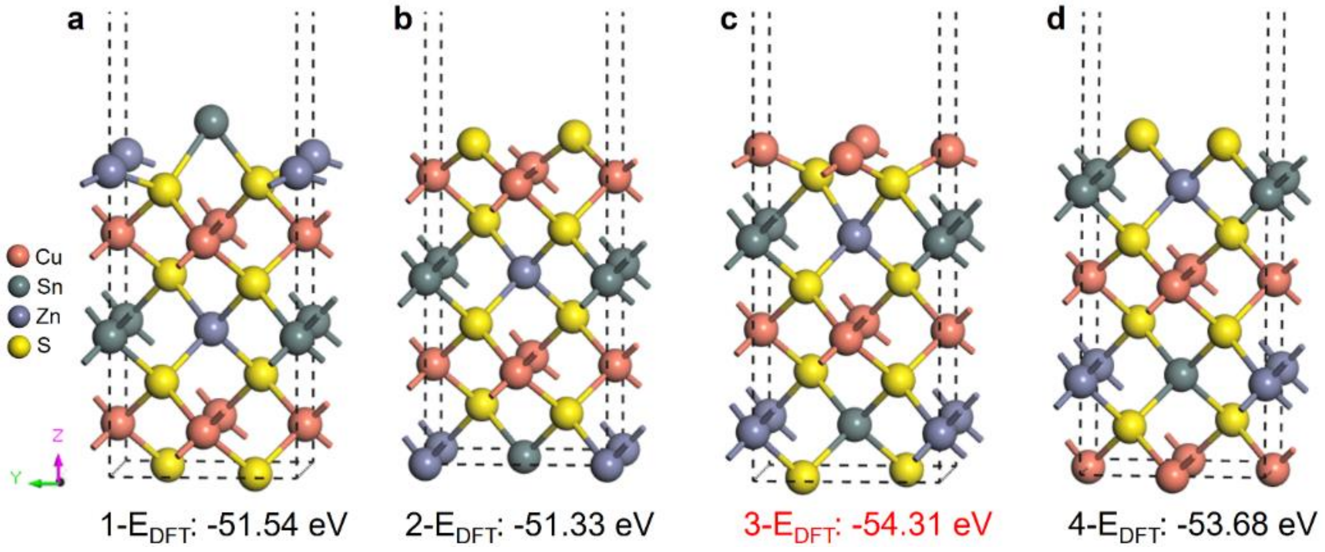

Supplementary Figure 2. The theoretical model and calculated total energy (E_{DFT}) of the pristine CZTS slab along (001) direction with different exposed atoms. (a) Sn-Zn surface. (b) S-Cu surface. (c) Cu-S surface. (d) S-Sn surface. The third model (c) is considered as the most stable structure with a surface energy of 0.748 J/m^2.

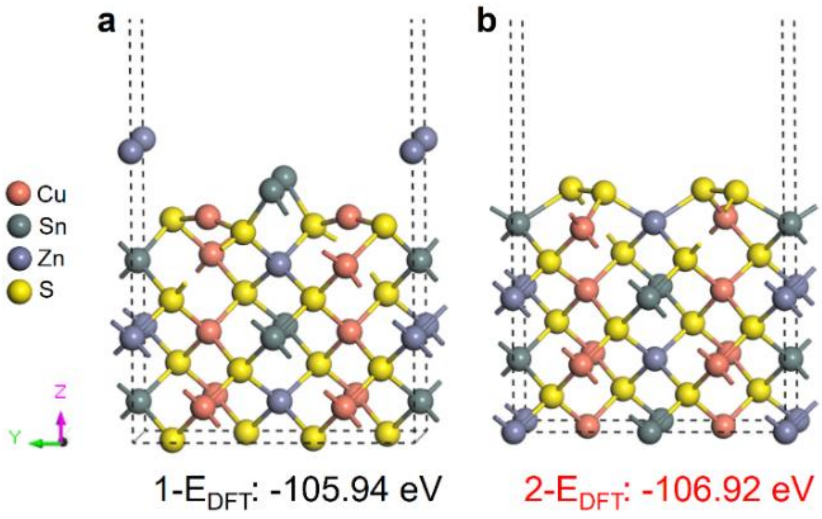

Supplementary Figure 3. The theoretical model and calculated total energy (E_{DFT}) of the pristine CZTS slab along (100) direction with different exposed atoms. (a) Zn-Sn surface. (b) S-Sn surface. The second model (b) is considered as the most stable structure with a surface energy of 0.866 J/m^2.

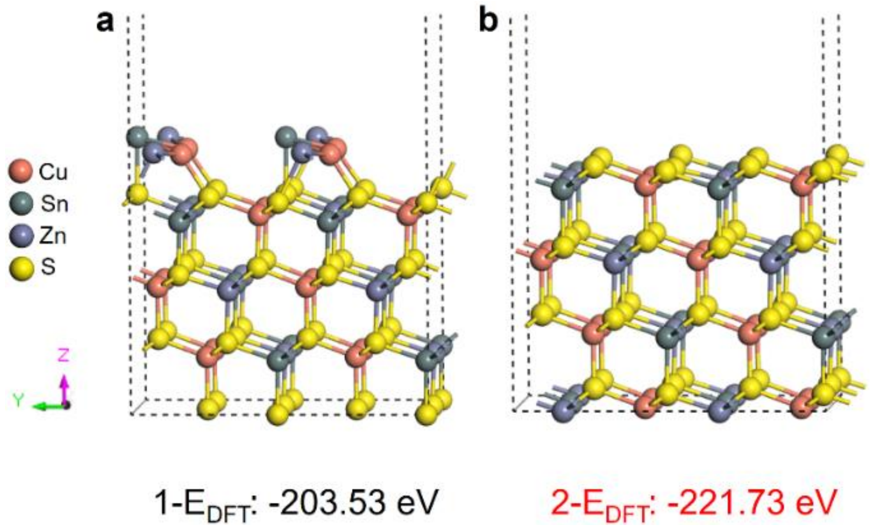

Supplementary Figure 4. The theoretical model and calculated total energy (E_{DFT}) of the pristine CZTS slab along (112) direction with different exposed atoms. (a) Cu-Zn-Sn surface. (b) S surface (referred to as 112-S). The second model (b) is considered as the most stable structure with a surface energy of 0.719 J/m^2.

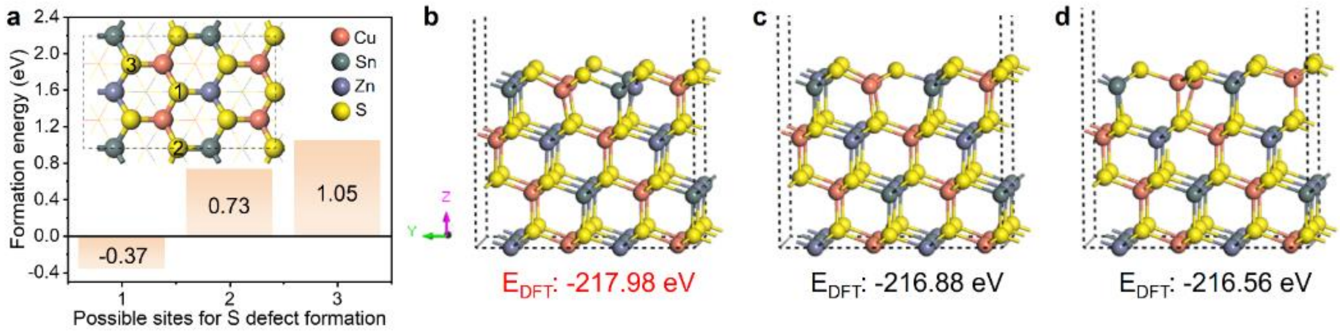

Supplementary Figure 5. The theoretical model of the optimized CZTS slab. (a) The formation energy of S defect at different position in 2D CZTS slab (top view). The S-defect CZTS (CZTS-S_v) slab model with S defect at 1 (b), 2 (c) and 3 (d) position. The (b) model is considered as the most stable structure with an S-defect formation energy of -0.37 eV.

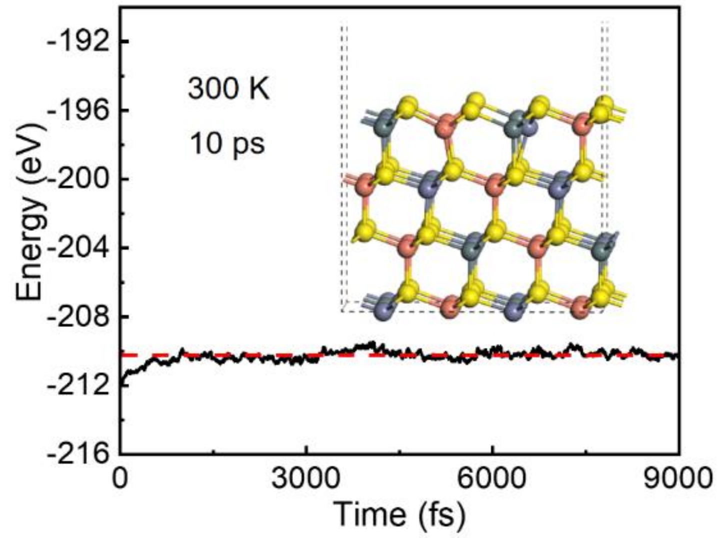

Supplementary Figure 6. MD simulation. The optimized CZTS-S_v slab at 300 K (relaxation time: 10 ps; time step: 3 fs).

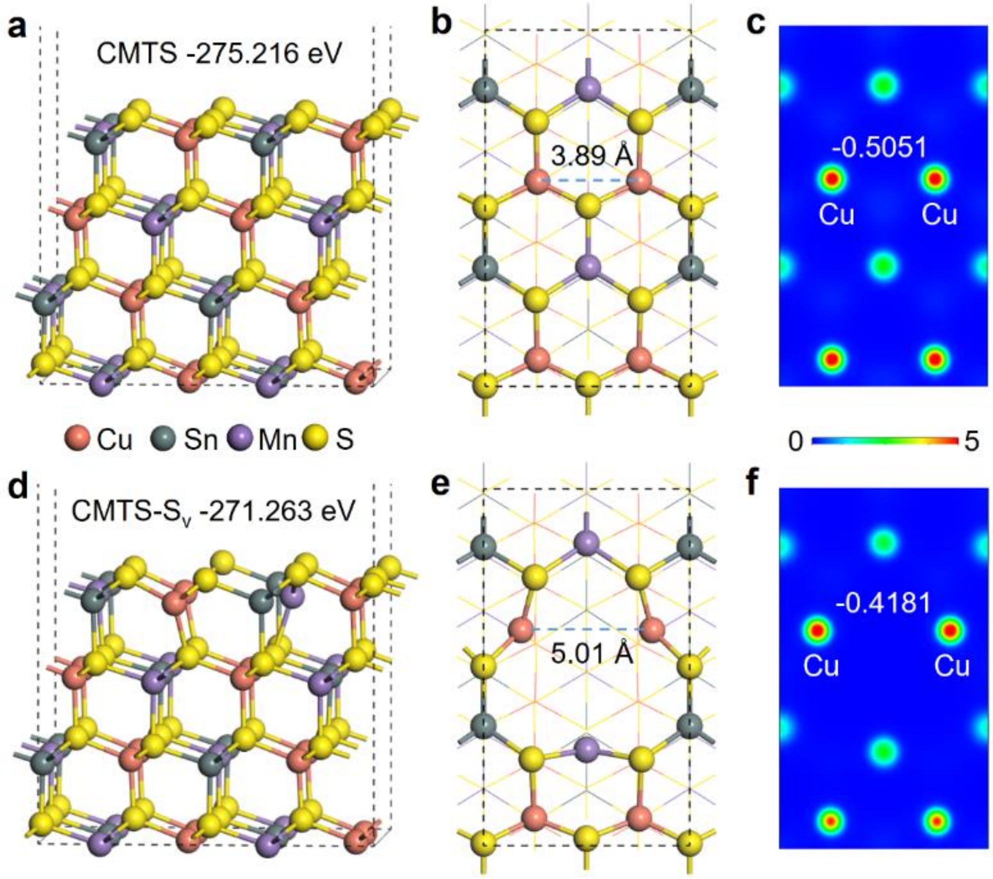

Supplementary Figure 7. The theoretical models, Cu-Cu bond length and the corresponding charge distribution with Bader charge of (a)-(c) Cu_2MnSnS_4 (CMTS) slab and (d)-(f) CMTS slab with S vacancy (S_v). The insert energy is the calculated total energy using DFT and the formation energy of S vacancy (E_{Sv}) is -0.163 eV.

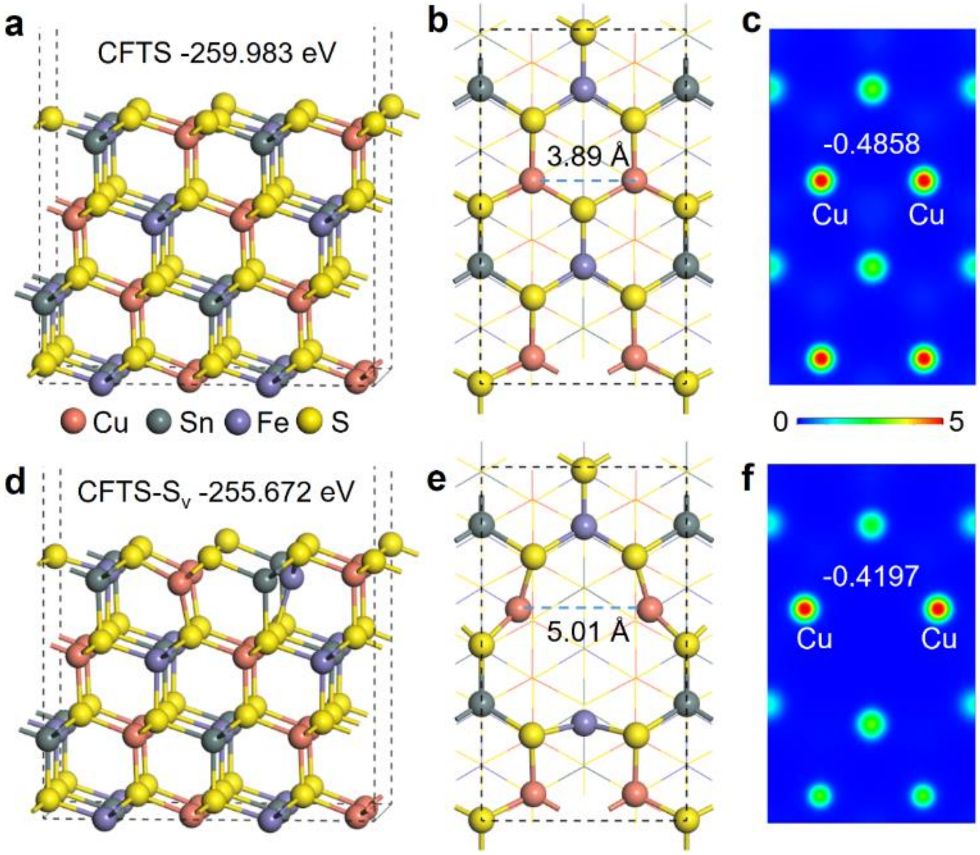

Supplementary Figure 8. The theoretical models, Cu-Cu bond length and the corresponding charge distribution with Bader charge of (a)-(c) Cu_2FeSnS_4 (CFTS) slab and (d)-(f) CFTS slab with S_v. The insert energy is the calculated total energy using DFT and E_{Sv} is 0.195 eV.

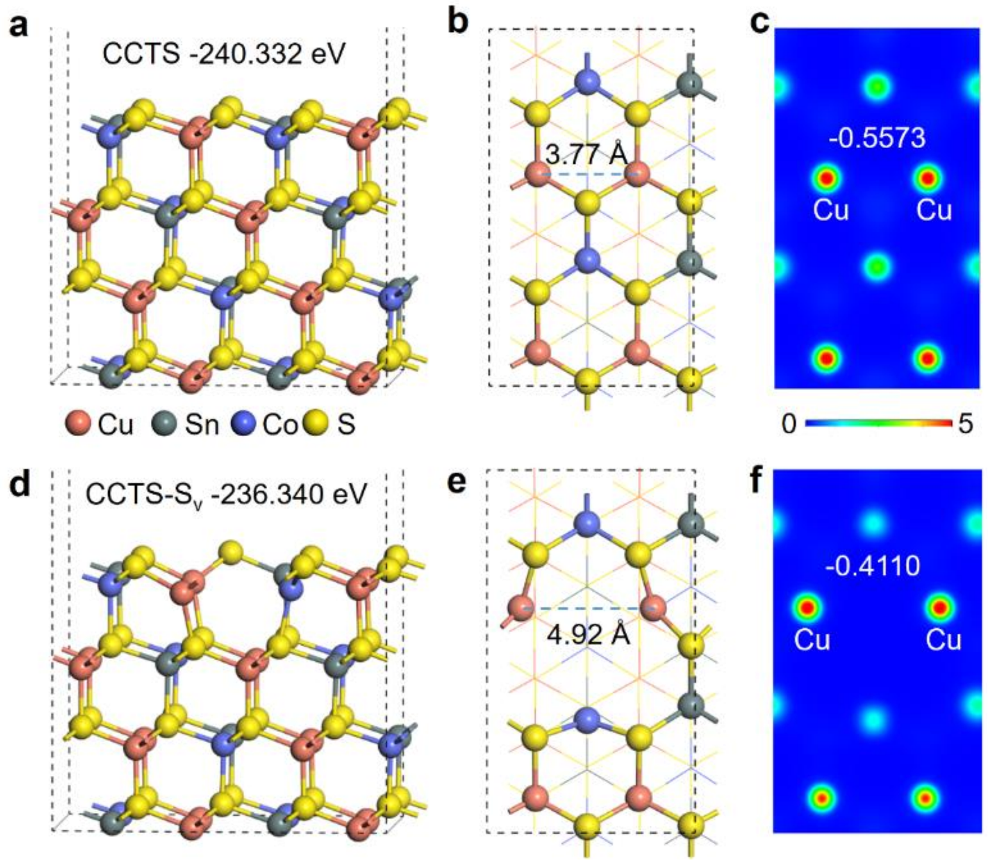

Supplementary Figure 9. The theoretical models, Cu-Cu bond length and the corresponding charge distribution with Bader charge of (a)-(c) Cu_2CoSnS_4 (CCTS) slab and (d)-(f) CCTS slab with S_v. The insert energy is the calculated total energy using DFT and E_{Sv} is -0.124 eV.

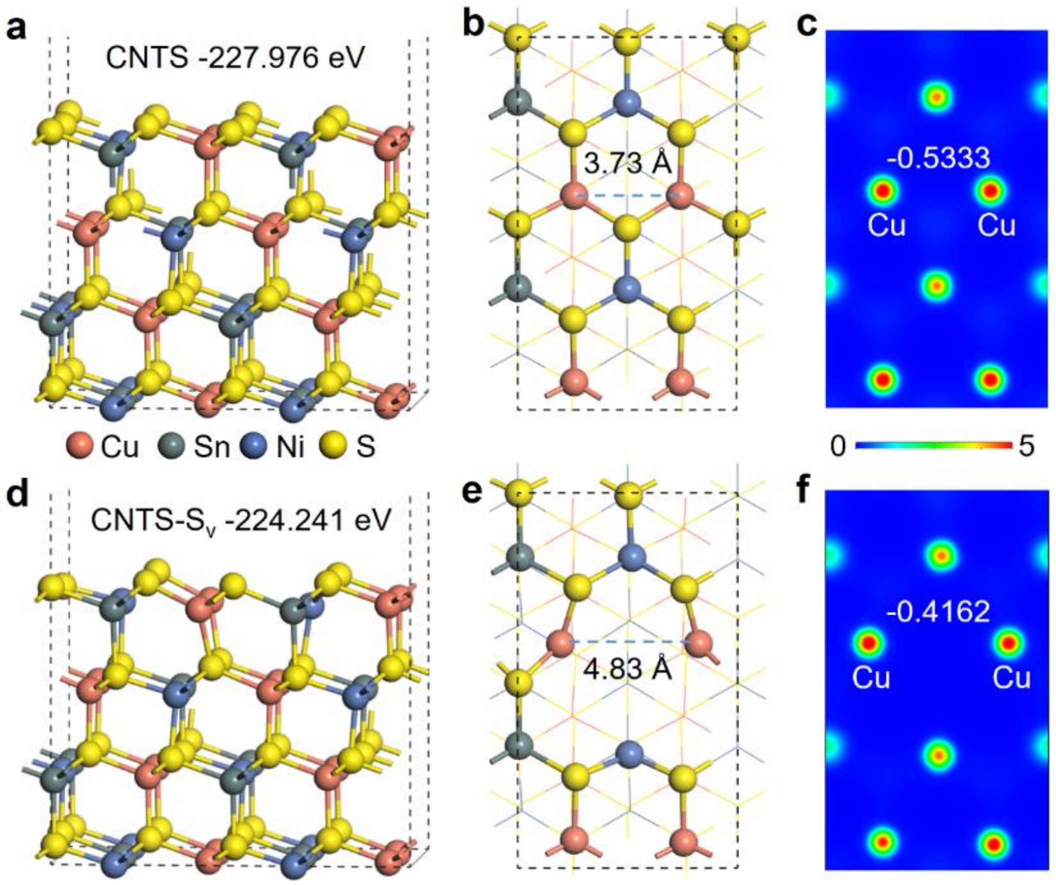

Supplementary Figure 10. The theoretical models, Cu-Cu bond length and the corresponding charge distribution with Bader charge of (a)-(c) Cu_2NiSnS_4 (CNTS) slab and (d)-(f) CNTS slab with S_v. The insert energy is the calculated total energy using DFT and E_{Sv} is -0.381 eV.

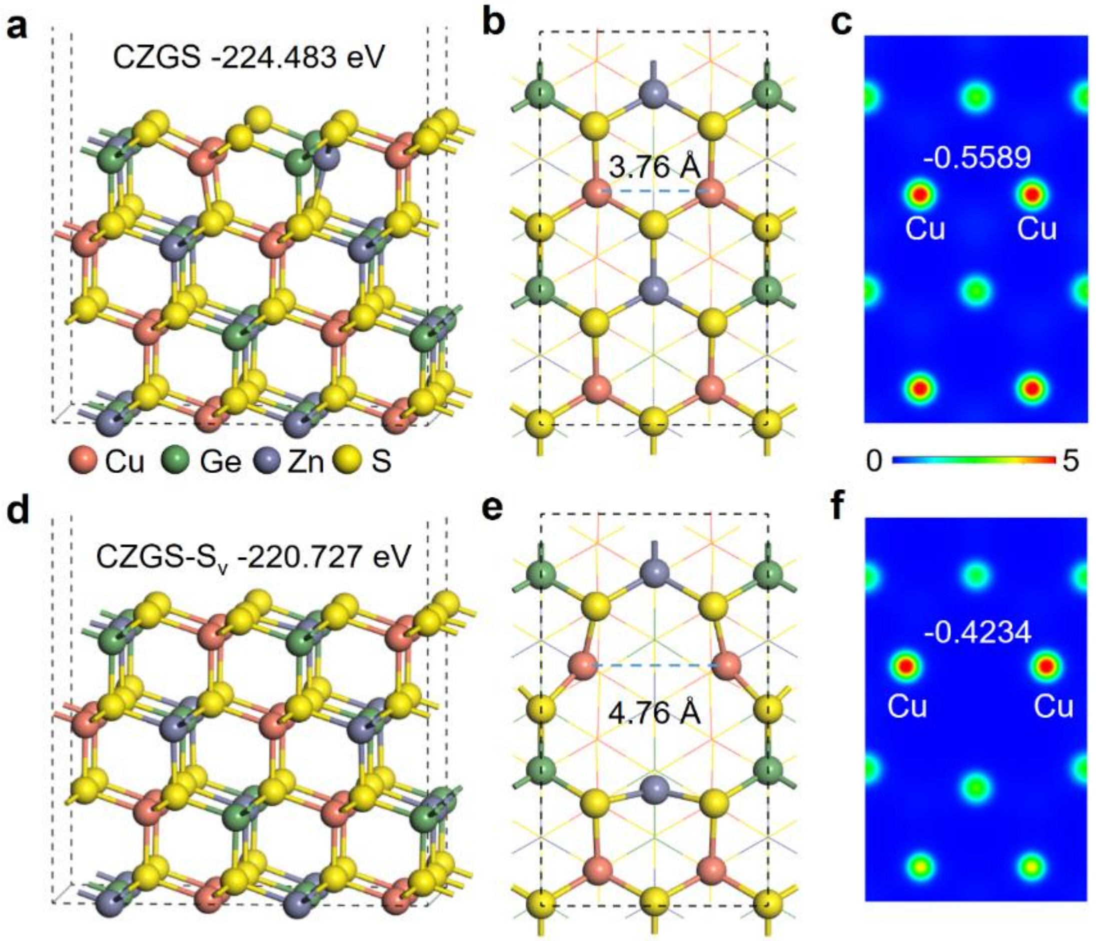

Supplementary Figure 11. The theoretical models, Cu-Cu bond length and the corresponding charge distribution with Bader charge of (a)-(c) Cu_2ZnGeS_4 (CZGS) slab and (d)-(f) CZGS slab with S_v. The insert energy is the calculated total energy using DFT and E_{Sv} is -0.360 eV.

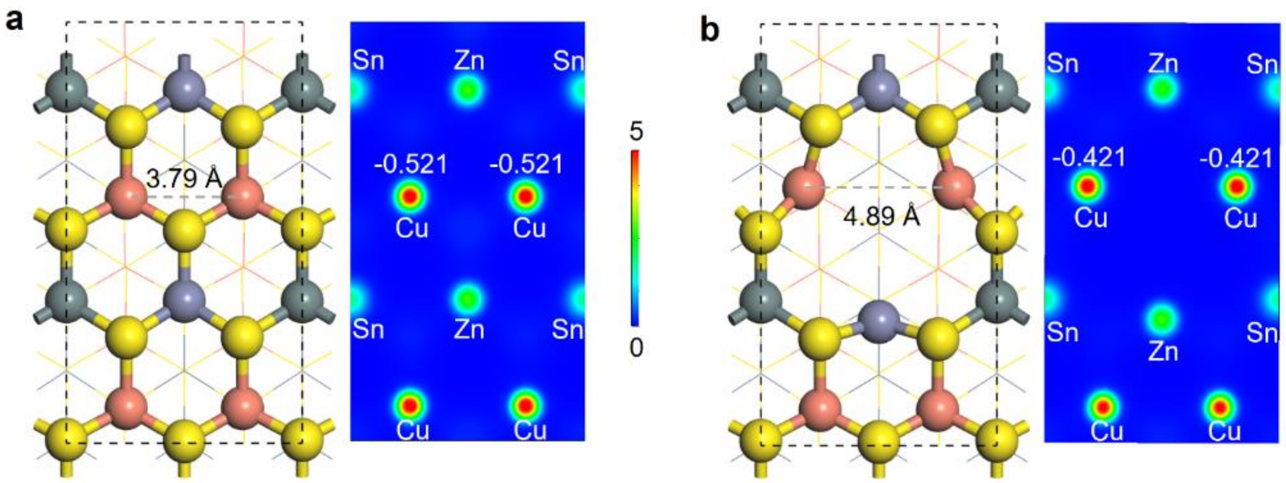

Supplementary Figure 12. DFT calculations for the pristine CZTS slab and the CZTS-S_v slab. The surface atom configuration, Cu-Cu bond length and Bader charge of (a) the pristine CZTS slab and (b) the CZTS-S_v slab, in which the negative value of Bader charge means the number of electrons lost from an atom.

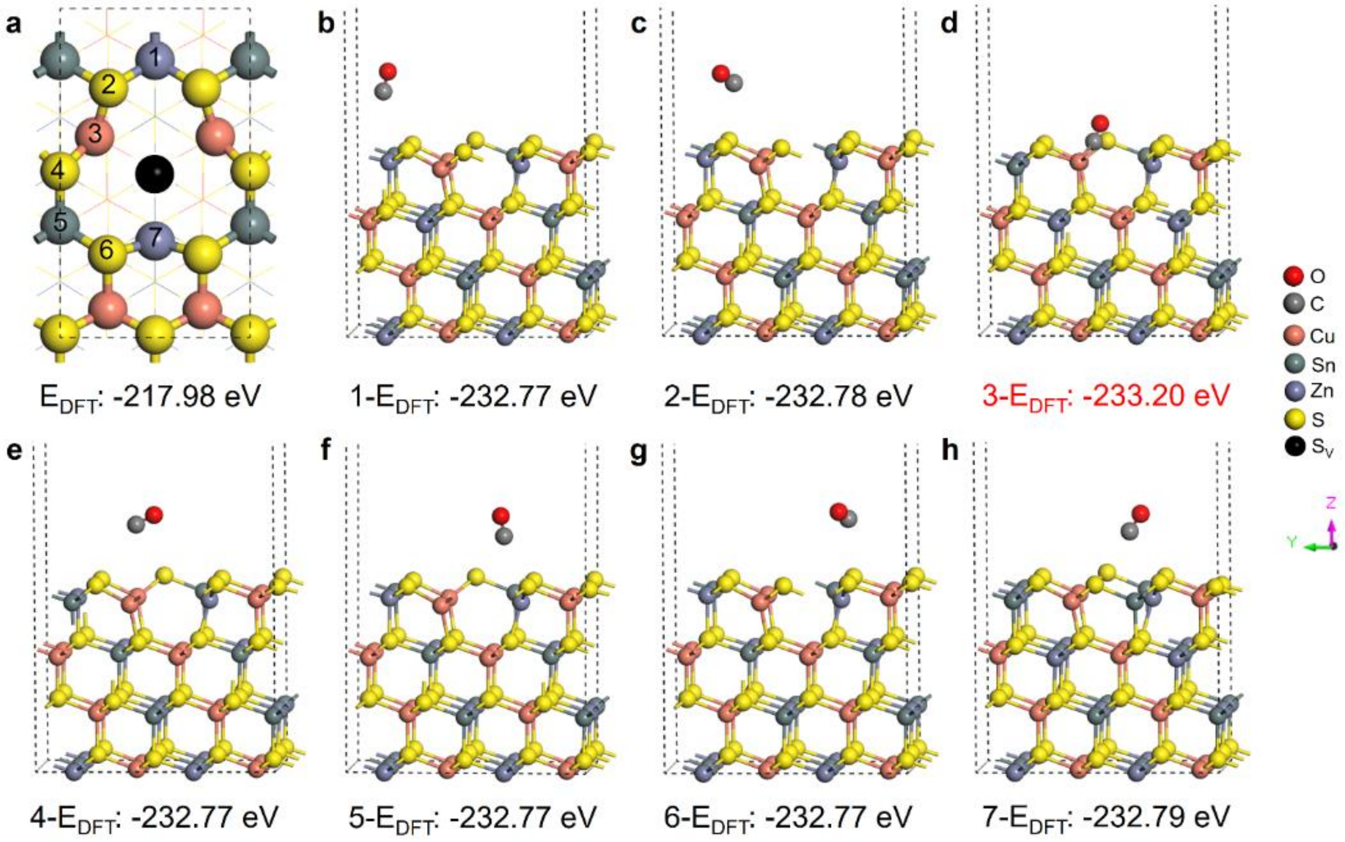

Supplementary Figure 13. The theoretical models of CO adsorption on the CZTS-S_v slab. (a) The possible adsorption sites (top view). (b)-(h) The CO adsorption models and calculated total energy (E_{DFT}) for CO adsorbed at 1-7 sites. The third (d) model is considered as the most stable adsorption structure with a CO adsorption energy of -0.43 eV.

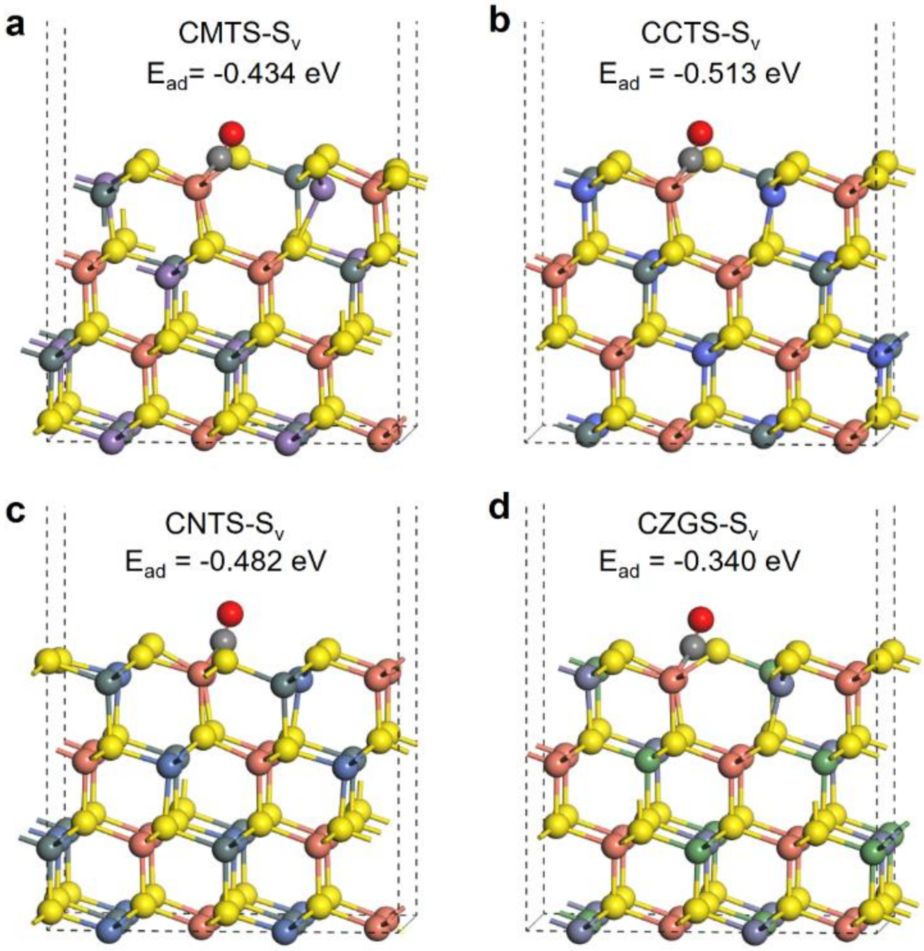

Supplementary Figure 14. The theoretical models and corresponding CO* adsorption energy of (a) CMTS-S_v, (b) CCTS-S_v, (c) CNTS-S_v, and (d) CZGS-S_v. E_{ad} is the calculated CO* adsorption energy.

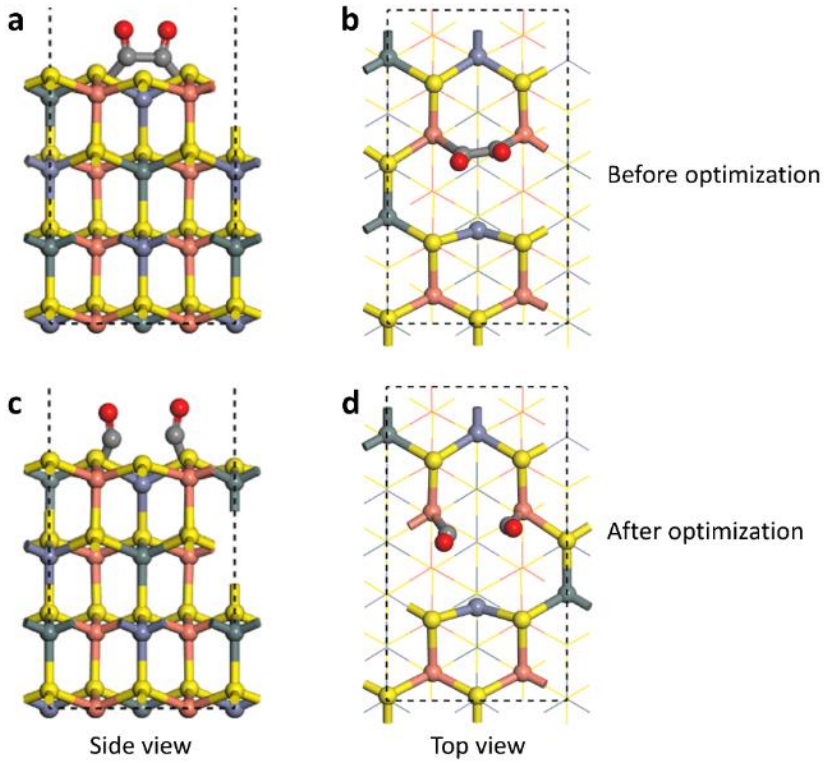

Supplementary Figure 15. C-C coupling process through CO-CO pathway. (a) Side view and (b) top view of the structure before optimization. (c) Side view and (d) top view of the structure after optimization.

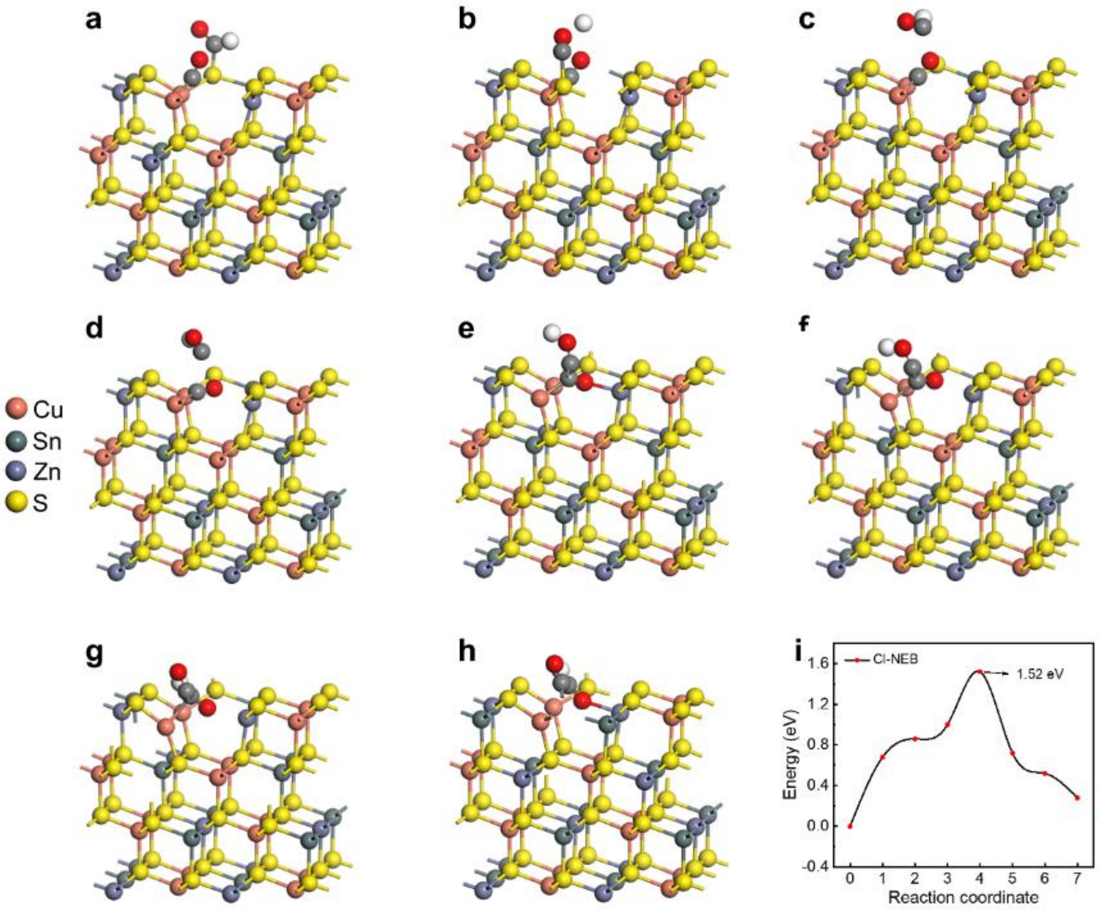

Supplementary Figure 16. C-C coupling process through CO-CHO pathway. (a)-(h) The TS models. (i) The calculated TS energy plots. The TS are calculated by the CI-NEB method, in which the energy barrier is computed to 1.52 eV.

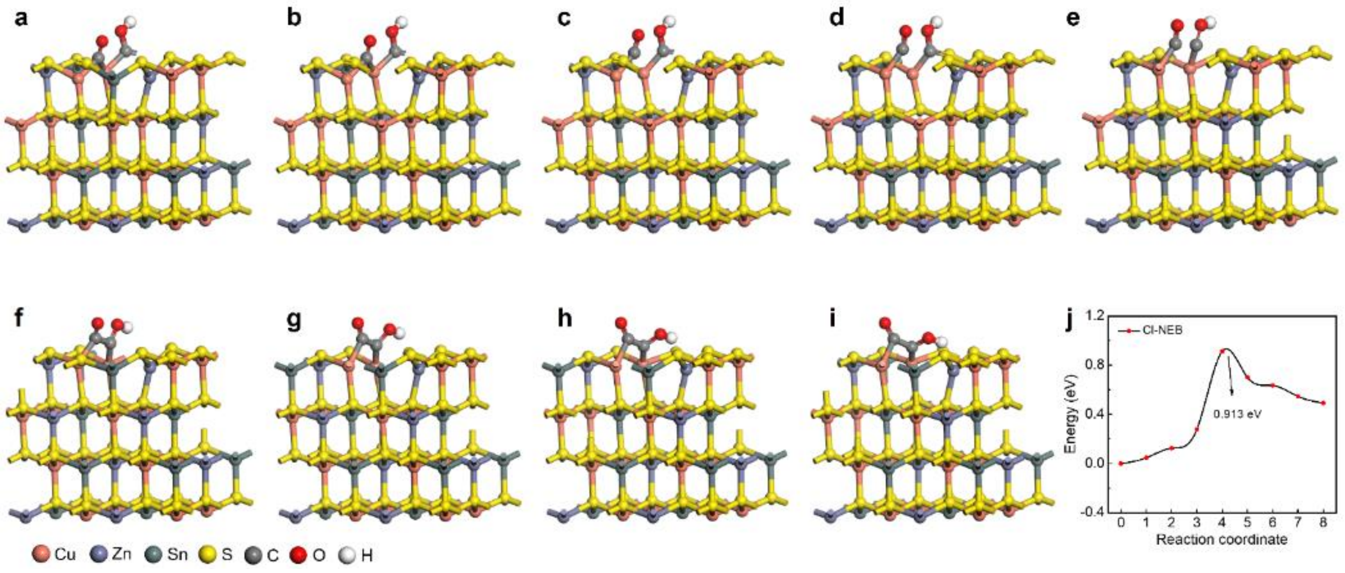

Supplementary Figure 17. Translation states (TS) of the coupling processes of CO* and COH* intermediates on the surface of the CZTS-S_v slab. (a)-(i) The TS models. (j) The calculated TS energy plots. The TS are calculated by the climbing image nudged elastic band (CI-NEB) method, in which the energy barrier is computed to 0.913 eV.

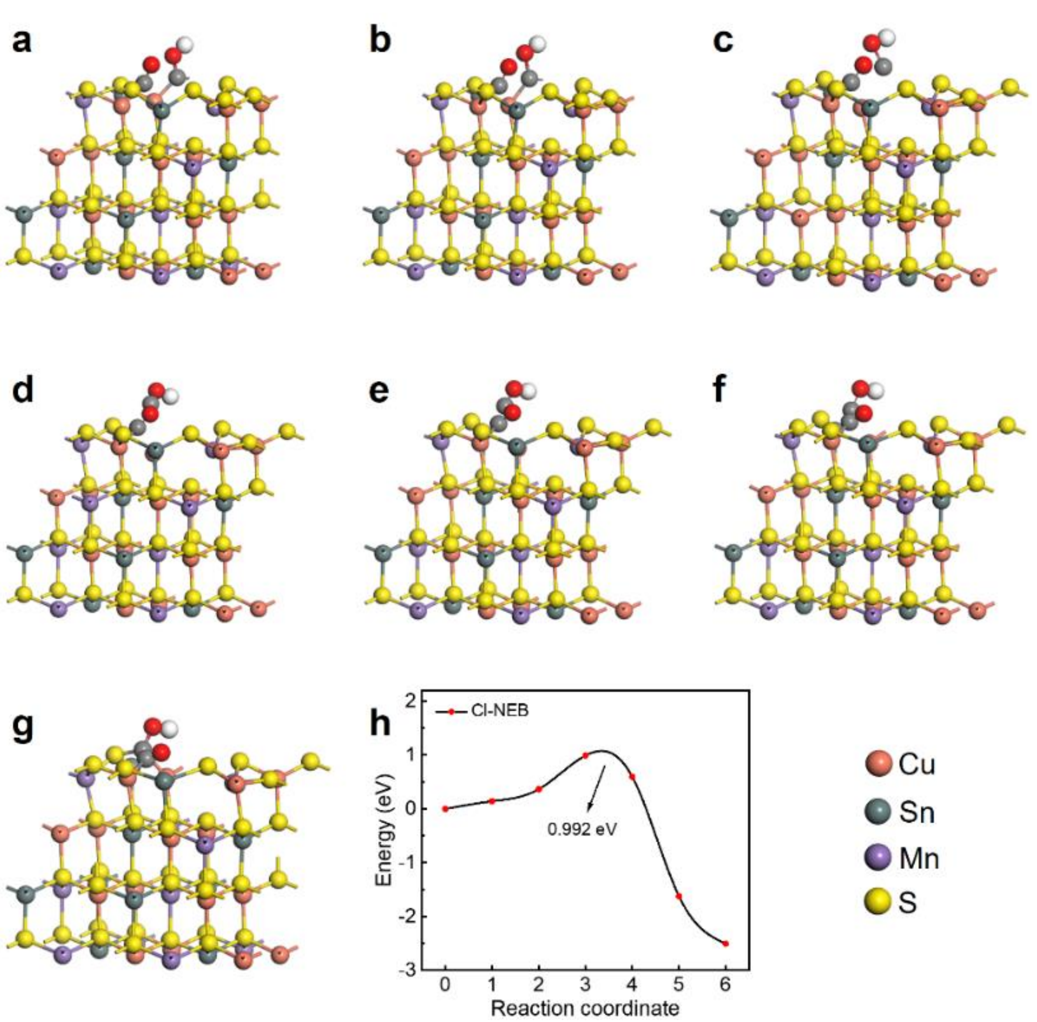

Supplementary Figure 18. TS of the coupling processes of CO* and COH* intermediates on the surface of the CMTS-S_v slab. (a)-(g) The TS models. (h) The calculated TS energy plots. The TS are calculated by the CI-NEB method, in which the energy barrier is computed to 0.992 eV.

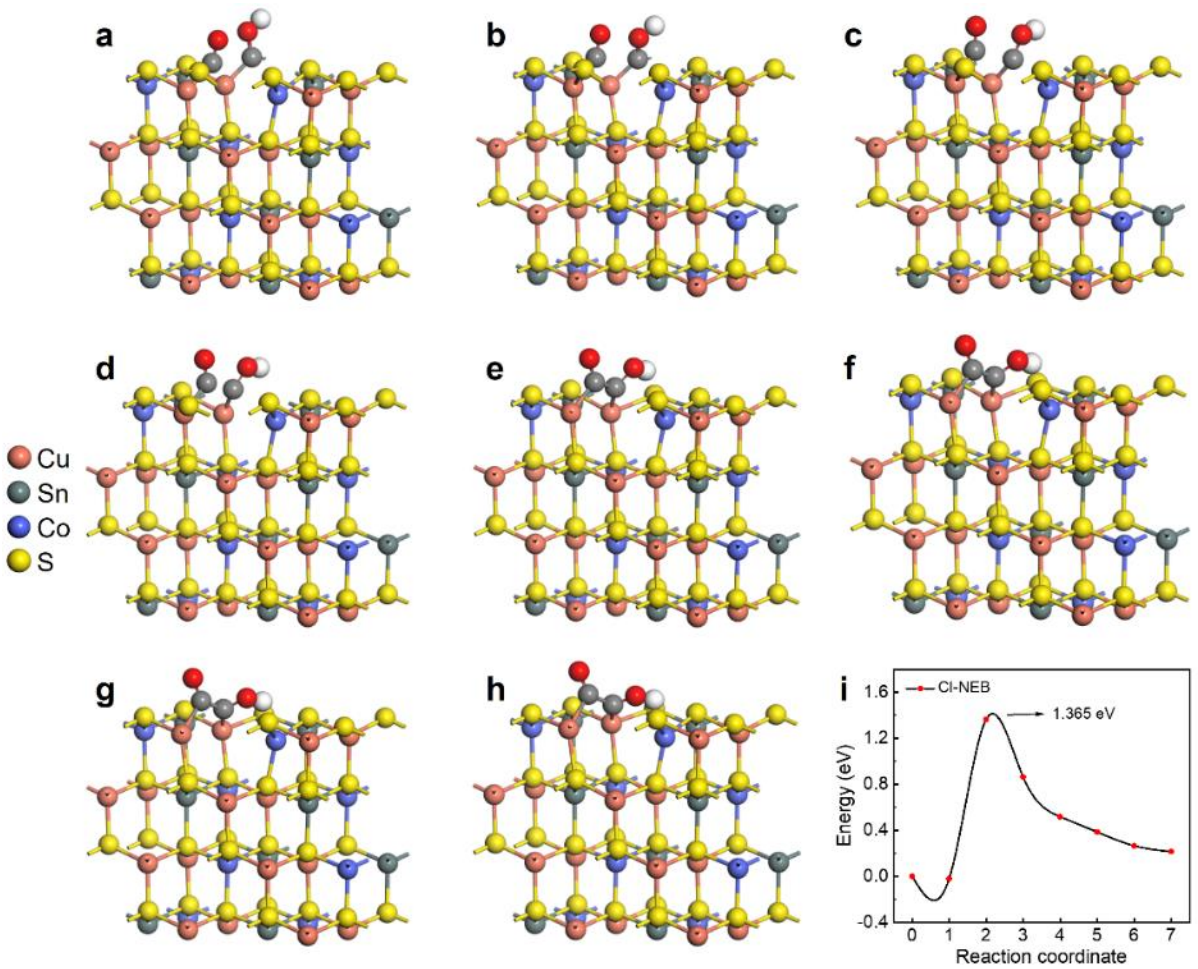

Supplementary Figure 19. TS of the coupling processes of CO* and COH* intermediates on the surface of the CCTS-S_V slab. (a)-(h) The TS models. (i) The calculated TS energy plots. The TS are calculated by the CI-NEB method, in which the energy barrier is computed to 1.365 eV.

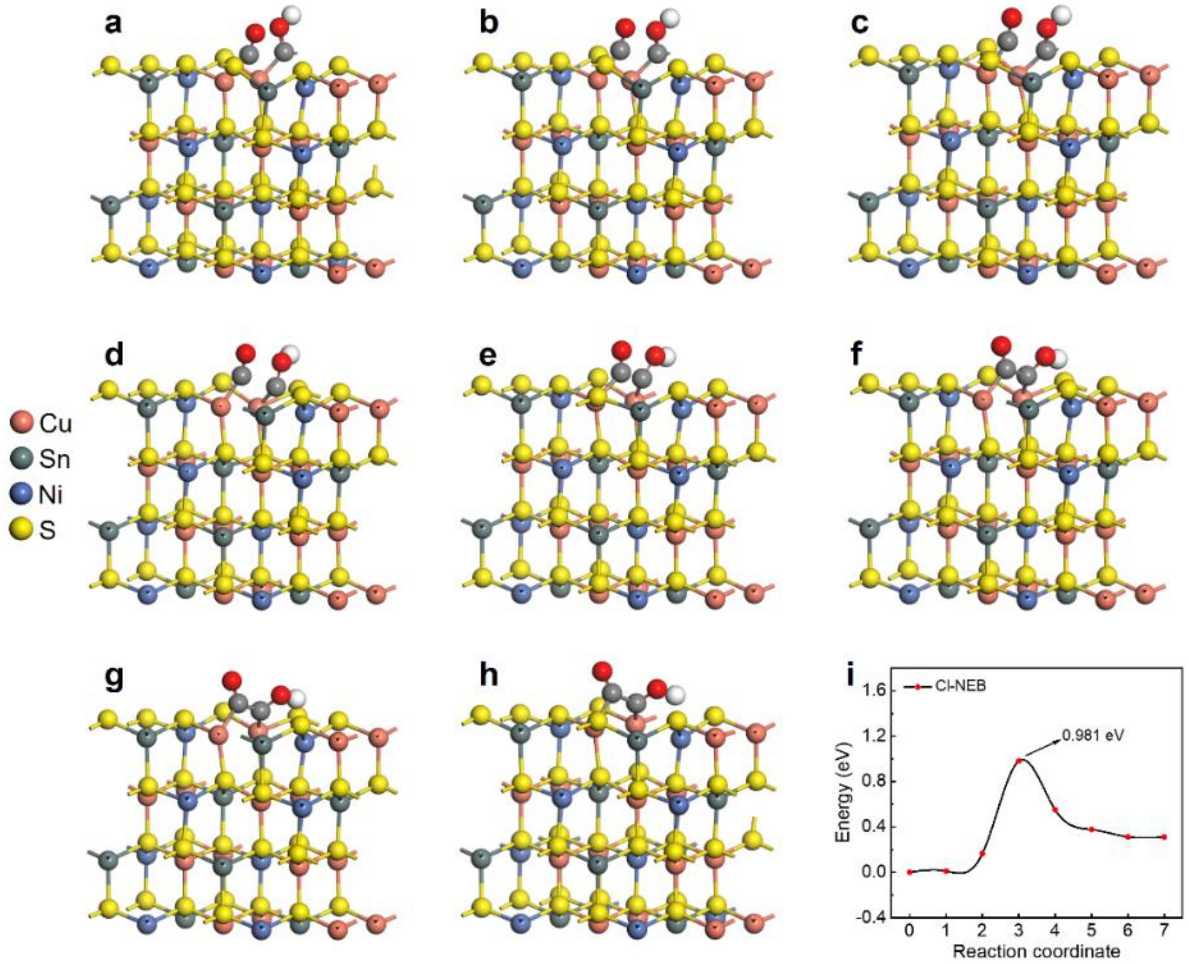

Supplementary Figure 20. TS of the coupling processes of CO* and COH* intermediates on the surface of the CNTS-S_v slab. (a)-(h) The TS models. (i) The calculated TS energy plots. The TS are calculated by the CI-NEB method, in which the energy barrier is computed to 0.981 eV.

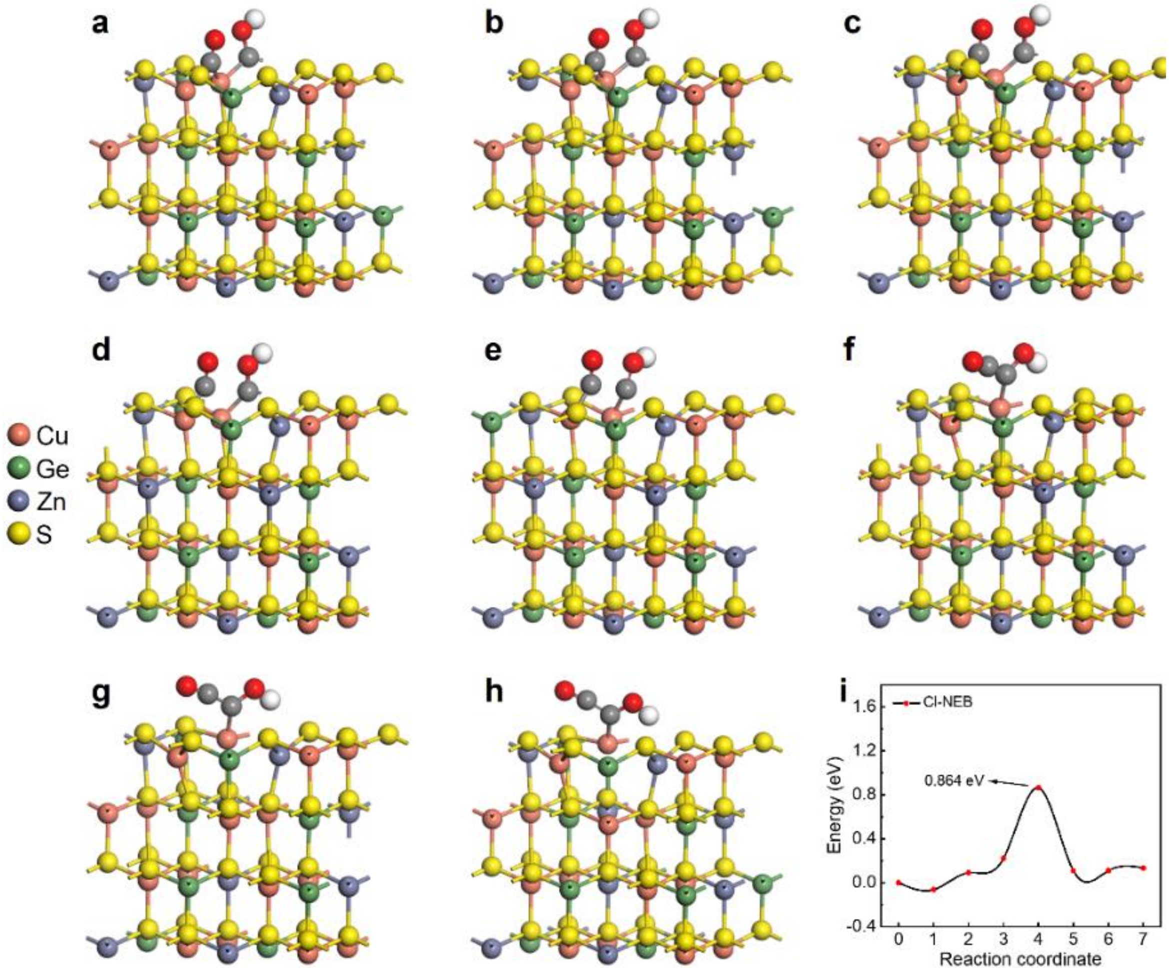

Supplementary Figure 21. TS of the coupling processes of CO* and COH* intermediates on the surface of the CZGS-S_V slab. (a)-(h) The TS models. (i) The calculated TS energy plots. The TS are calculated by the CI-NEB method, in which the energy barrier is computed to 0.864 eV.

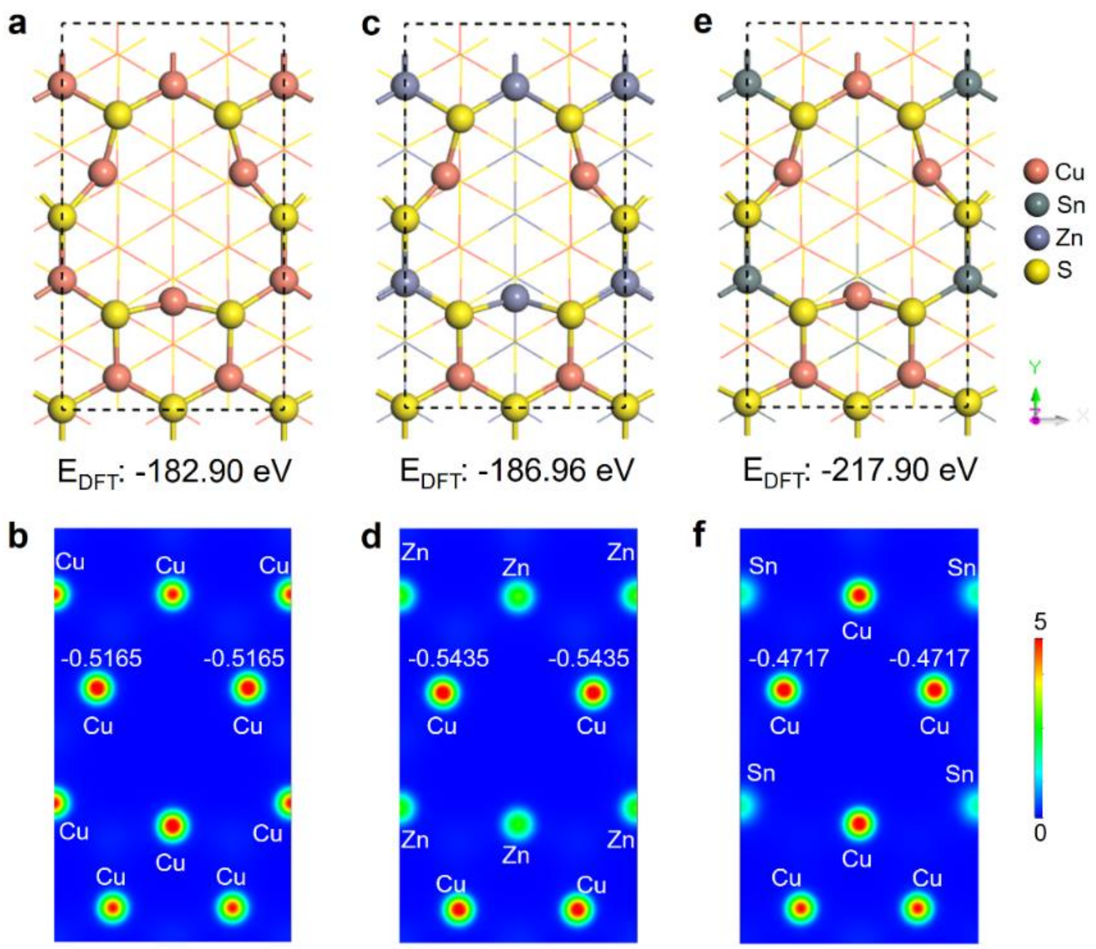

Supplementary Figure 22. The theoretical models and the corresponding charge distribution with Bader charge of (a)-(b) CuS-S_v slab, (c)-(d) CuZnS-S_v slab and (e)-(f) Cu_3SnS_4-S_v slab. E_{DFT} is the calculated total energy of the slab model.

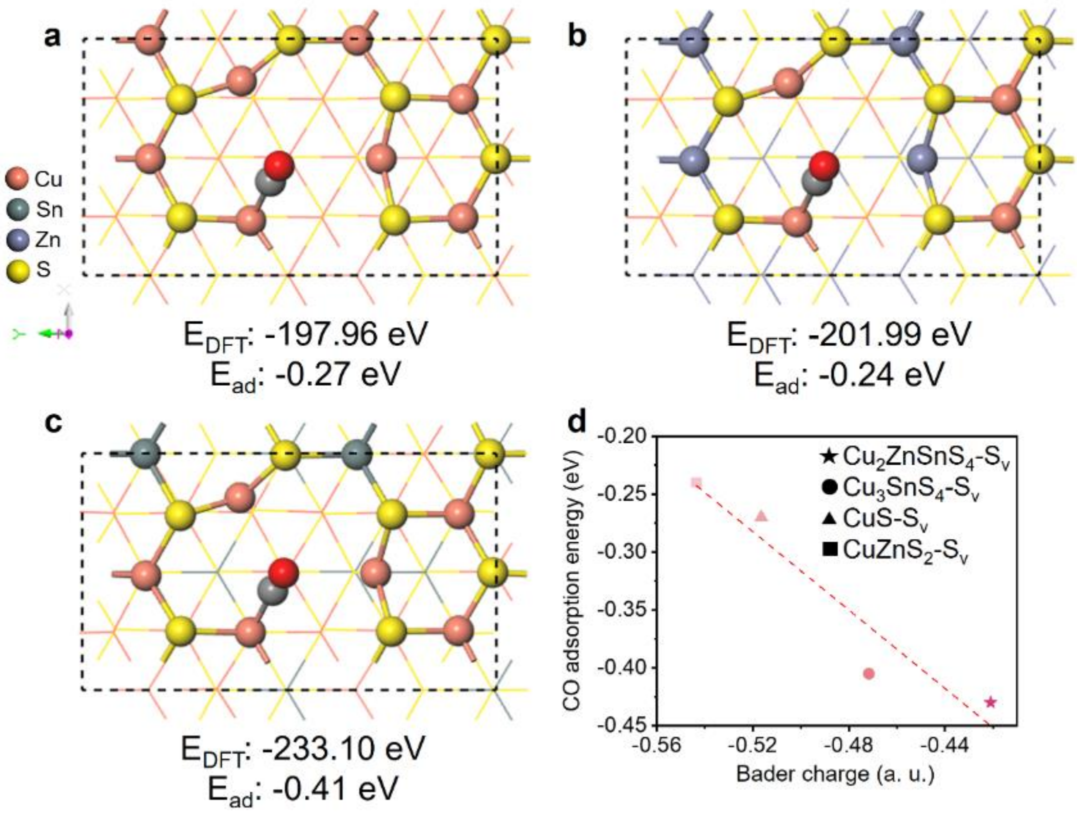

Supplementary Figure 23. The theoretical models of CO intermediate adsorbed on (a) CuS-S_v slab, (b) CuZnS-S_v and (c) Cu_3SnS_4-S_v slab. (d) E_{ad}(CO) over similar surfaces with different Cu, Zn, Sn, S elements. E_{DFT} is the calculated total energy of the slab model, E_{ad} represents the corresponding CO adsorption energy.

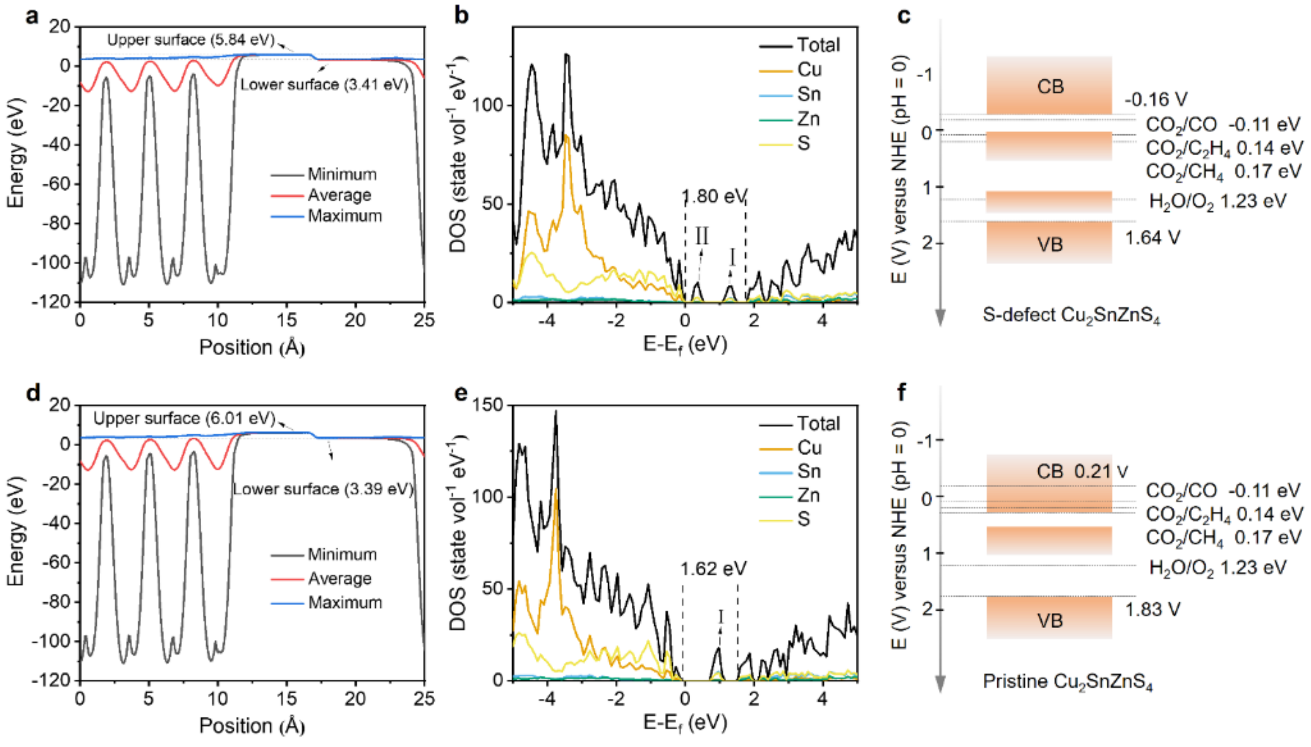

Supplementary Figure 24. The theoretical band structure of the CZTS-S_V slab and pristine CZTS slab. (a) The electrostatic potential of CZTS-S_V slab. (b) The calculated DOS of CZTS-S_V slab, in which I and II are attributed to the surface states and defect states, respectively. (c) Theoretical band structure and band edge position of CZTS-S_V slab. (d) The electrostatic potential of pristine CZTS slab. (e) The calculated DOS of pristine CZTS slab, in which I is attributed to the surface states. (f) Theoretical band structure and band edge position of pristine CZTS slab.

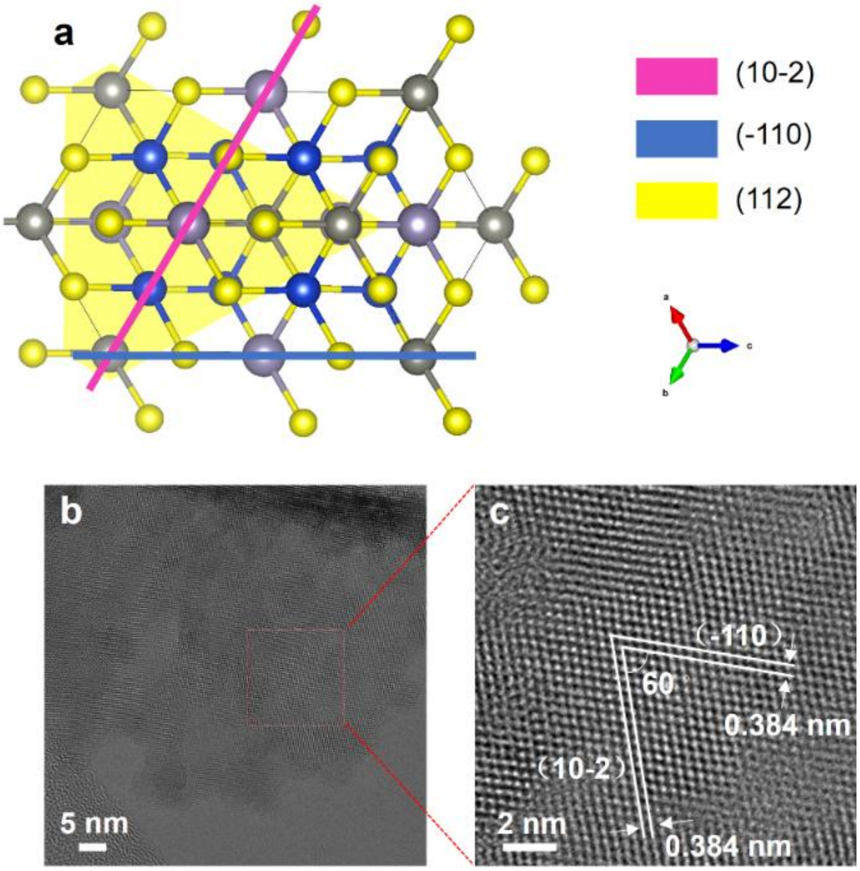

Supplementary Figure 25. Crystal structure of CZTS-S_v nanosheet. (a) The theoretical models of CZTS nanosheet along [112] direction. (b)-(c) The HRTEM images of the experimentally synthesized CZTS-S_v nanosheet, in which the exposed facet can be inferred along [112] direction because 0.384 nm interplanar distances match well with the d_{-110} and d_{10-2} spacings, and the corresponding dihedral angle of 60° agrees well with the calculated angle between the (-110) and (10-2) planes. For HRTEM images of CMTS in Figure 2a, the exposed facet can be inferred along [112] direction because 0.389 nm and 0.386 nm interplanar distances match well with the d_{-110} and d_{10-2} spacings, and the corresponding dihedral angle of 60° agrees well with the calculated angle between the (-110) and (10-2) planes. For HRTEM images of CCTS in Figure 2b, the exposed facet can be inferred along [112] direction because 0.382 nm interplanar distances match well with the d_{-110} and d_{10-2} spacings, and the corresponding dihedral angle of 60° agrees well with the calculated angle between the (-110) and (10-2) planes. For HRTEM images of CNTS in Figure 2a, the exposed facet can be inferred along [111] direction because 0.384 nm interplanar distances match well with the d_{-110} and d_{10-2} spacings, and the corresponding dihedral angle of 60° agrees well with the calculated angle between the (110) and (101) planes.

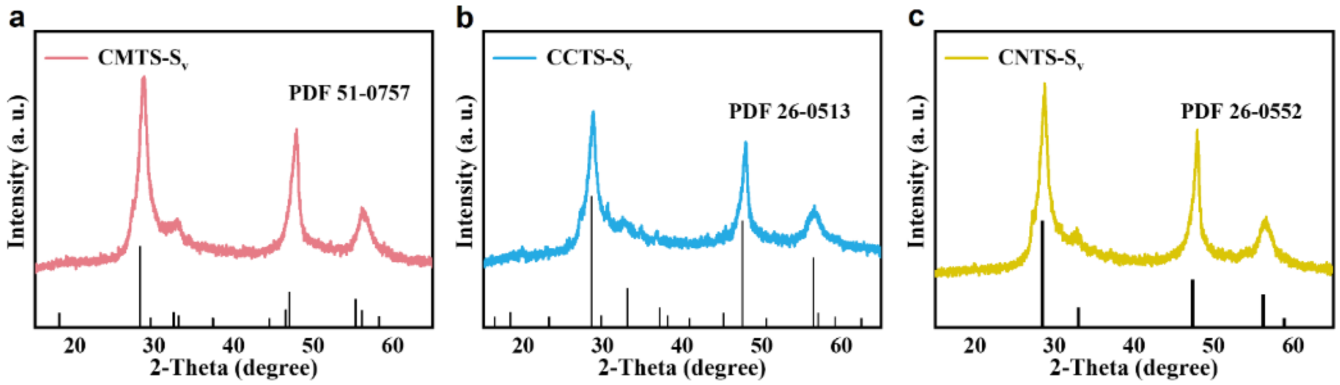

Supplementary Figure 26. The experimental characterization of $Cu_2M_iM_jS_4$-S_v. XRD patterns for the synthesized (a) CMTS-S_v nanosheet, (b) CCTS-S_v nanosheet and (c) CNTS-S_v nanosheet.

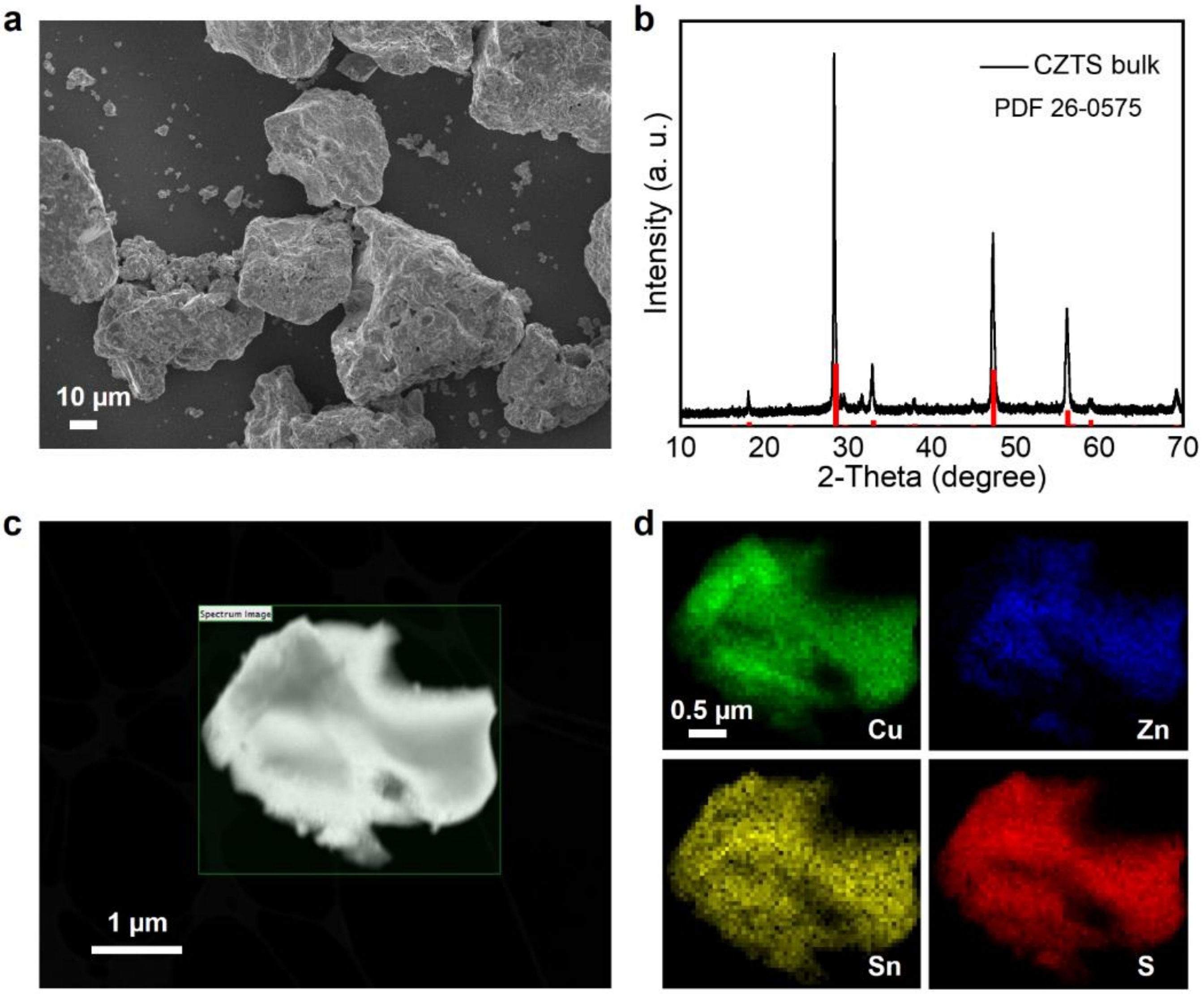

Supplementary Figure 27. The characterizations for CZTS bulk. (a) SEM images. (b) XRD pattern. (c)-(d) STEM and the corresponding EDS mapping images.

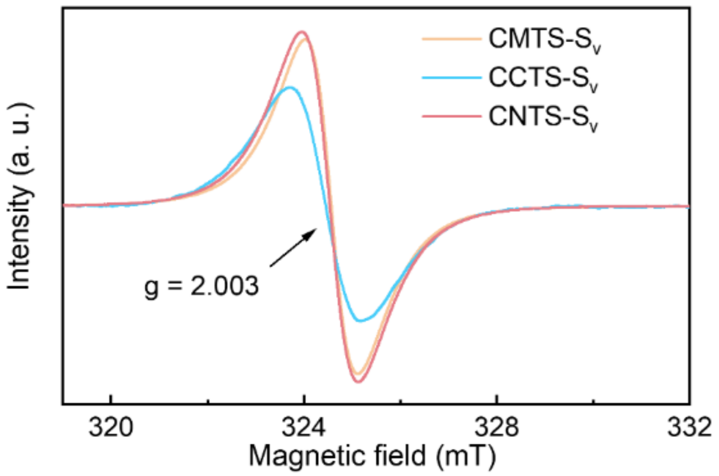

Supplementary Figure 28. ESR spectra for the synthesized CMTS-S_v nanosheet, CCTS-S_v nanosheet and CNTS-S_v nanosheet. The signal at g = 2.003 corresponds to the S vacancy.

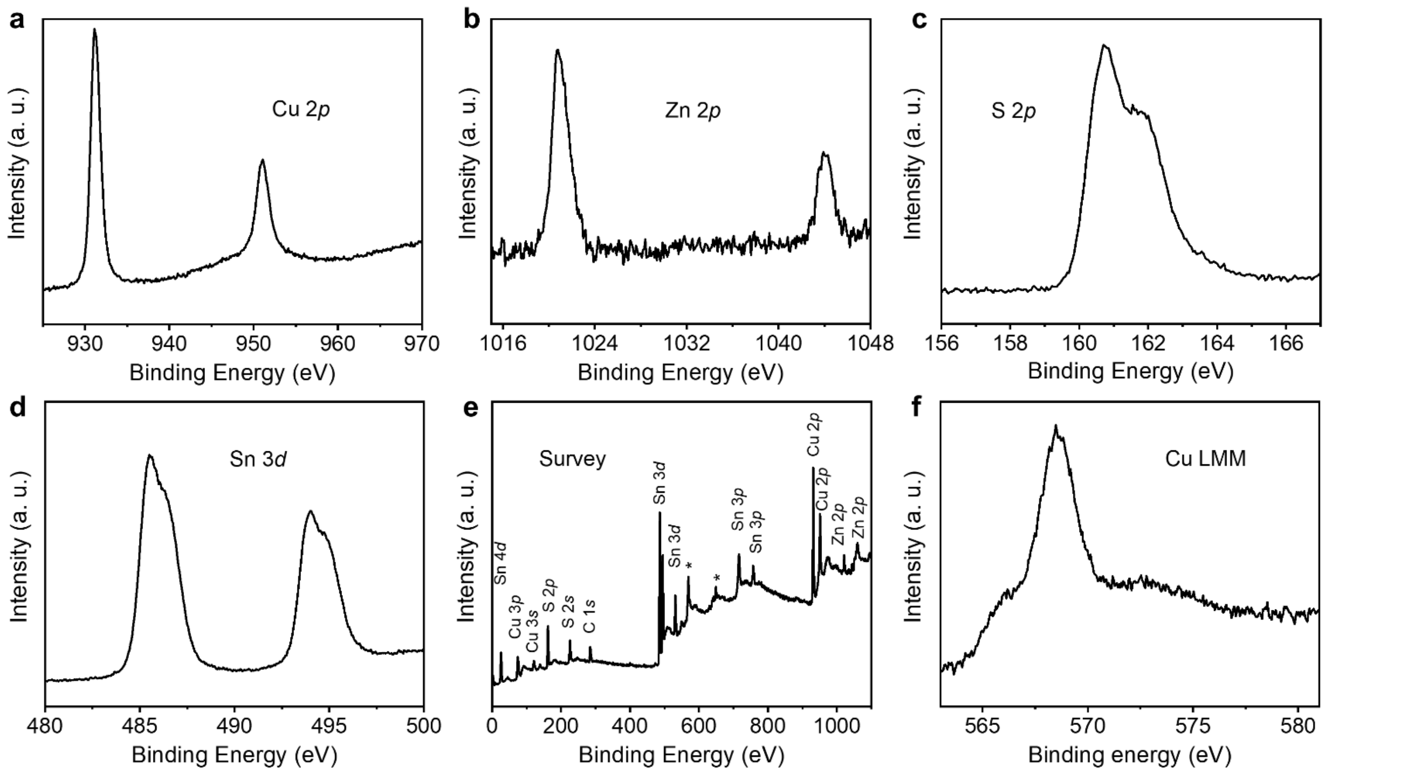

Supplementary Figure 29. XPS spectrum of CZTS bulk. (a) High-resolution Cu 2*p* spectra; (b) high-resolution Zn *p* spectra; (c) high-resolution S 2*p* spectra; (d) High-resolution Sn 3*d* spectra; (e) survey spectra; (f) high-resolution Cu LMM spectra.

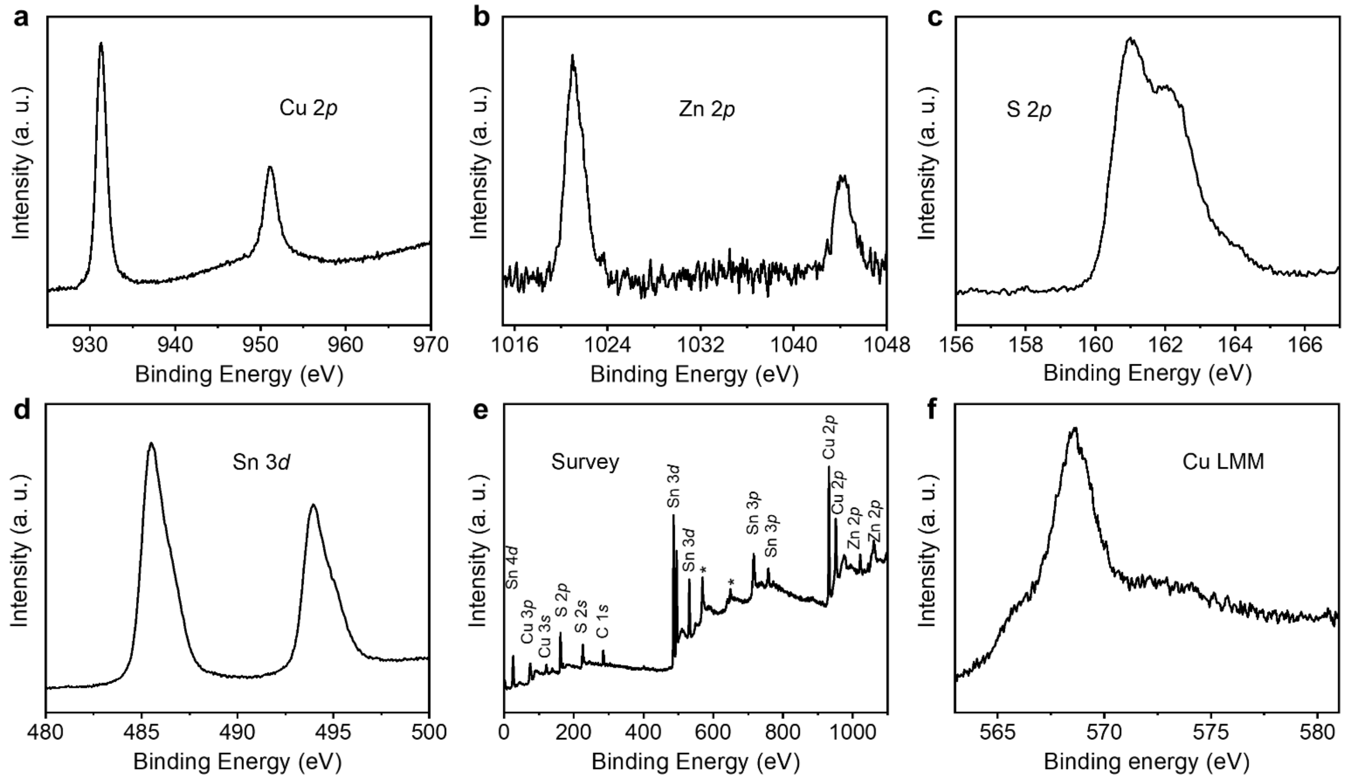

Supplementary Figure 30. XPS spectrum of CZTS-S_v nanosheet. (a) High-resolution Cu 2*p* spectra; (b) high-resolution Zn *p* spectra; (c) high-resolution S 2*p* spectra; (d) High-resolution Sn 3*d* spectra; (e) survey spectra; (f) high-resolution Cu LMM spectra.

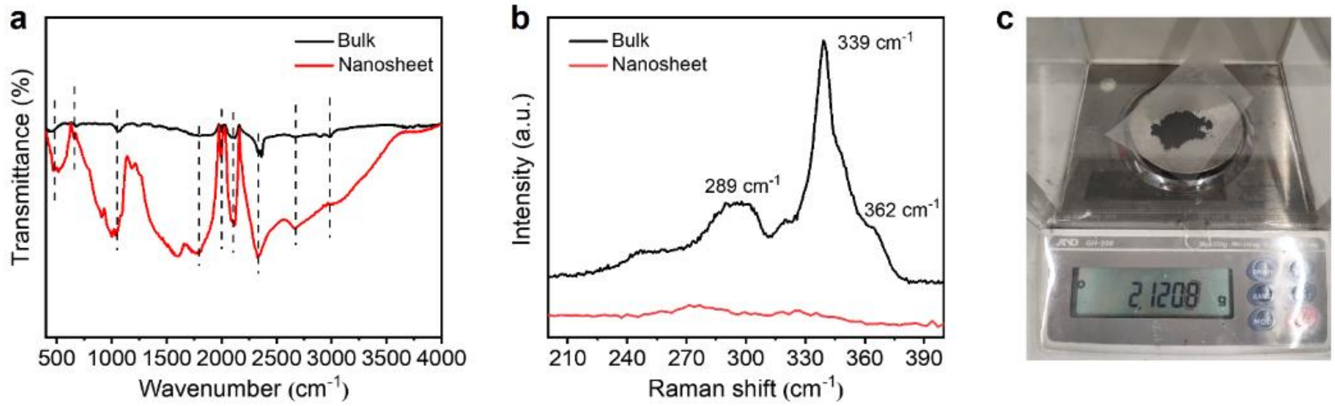

Supplementary Figure 31. Spectroscopy characterizations of CZTS bulk and nanosheet. (a) FT-IR and (b) Raman spectrum of CZTS bulk and nanosheets. The IR spectrum shows that nanosheet samples possess the same characteristic peaks with the bulk, and no obvious peaks of organics are observed, which indicated the final nanosheets are clean 2D free-standing CZTS-S_v sheets. And the CZTS-S_v nanosheet doesn't show obvious Raman peak, which confirms its poor crystallinity, indicating the presence of surface S defect. In the contrast, the Raman shifts at 289, 339 and 362 cm^{-2} were detected in the CZTS bulk, which are close to the previous reports. (c) The digital image of upscalable synthesis of CZTS nanosheet. Samples of more than 2 grams can be easily synthesized using the solvothermal method.

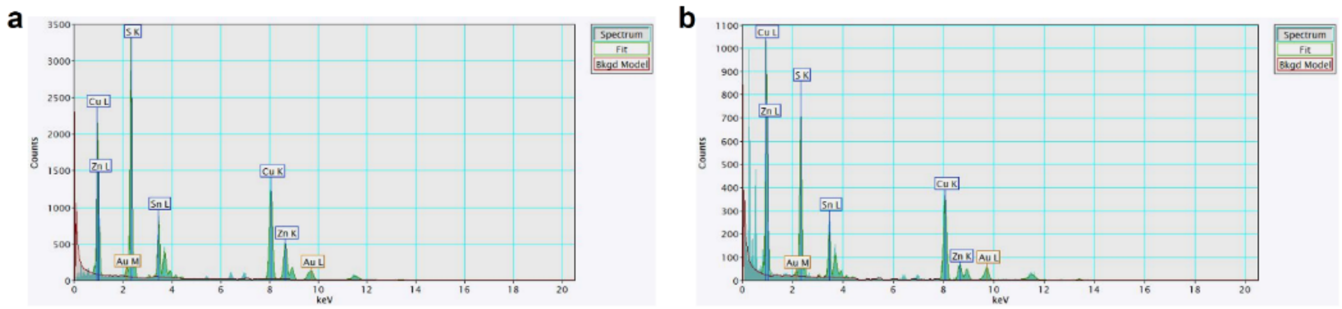

c

EDS element content of TEM									
	Element	Shell	Signal (Counts)	Fitting Mode	Comp. (at.%)	Comp. (wt.%)	k-Factor (/Si)	k-Factor Mode	Det. Corr.
Bulk	Cu	K	22.29e+03	Theoretical	26.44	28.38	1.794	Theoretical	1.00
	Zn	K	9.09e+03	Theoretical	11.09	12.25	1.900	Theoretical	1.00
	Sn	L	15.43e+03	Theoretical	17.45	35.00	3.195	Theoretical	0.97
	**S**	**K**	**32.32e+03**	**Theoretical**	**45.00**	**24.38**	**1.063**	**Theoretical**	**0.93**
Nanosheet	Cu	K	19.56e+03	Theoretical	32.70	33.28	1.794	Theoretical	1.00
	Zn	K	9.12e+03	Theoretical	15.66	16.35	1.900	Theoretical	1.00
	Sn	L	10.57e+03	Theoretical	16.84	32.30	3.195	Theoretical	0.97
	**S**	**K**	**17.72e+03**	**Theoretical**	**34.8**	**18.00**	**1.063**	**Theoretical**	**0.93**

Supplementary Figure 32. Energy dispersive spectroscopy (EDS) for CZTS bulk and nanosheet. (a) Bulk and (b) nanosheet, in which Au grids were used for these elements mapping and EDS tests; (c) the corresponding atomic percentage in CZTS bulk and nanosheet by TEM extended EDS. Herein, we take the content of S element as a reference for S defects.

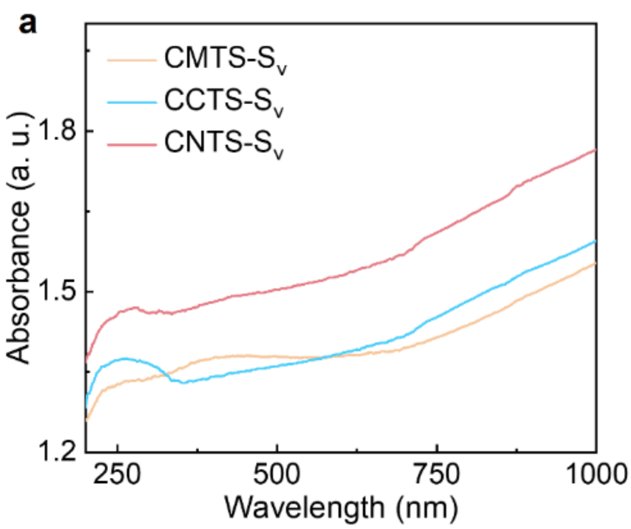

Supplementary Figure 33. UV-vis-NIR diffuse reflectance spectra measurements. (a) UV-vis-NIR diffuse reflectance spectra and (b) the corresponding Tauc plots for the synthesized CMTS-S_v nanosheet, CCTS-S_v nanosheet and CNTS-S_v nanosheet.

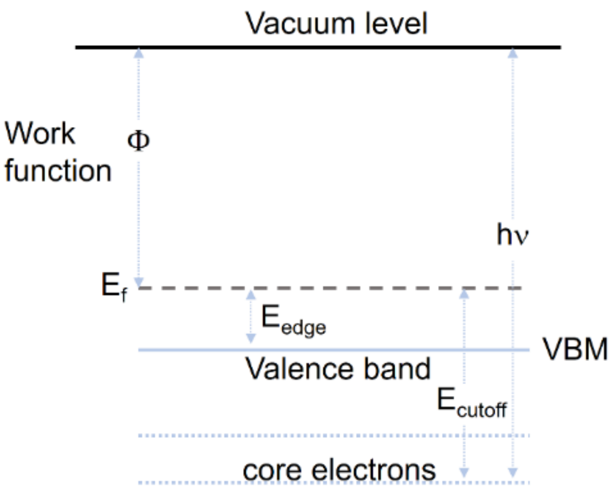

Supplementary Figure 34. Schematic illustration of the energy level information from UPS. The valence band maxima (VBM) of the samples referenced to Normal Hydrogen Electrode (NHE) can be obtained according to the following equations[17, 18]:

$$\Phi = h\nu - E_{cutoff}$$

$$E_{VBM} = E_{edge} + \Phi - 4.5 \text{ (} vs \text{ NHE, pH = 0)}$$

where Φ is the work function, hν is the photon energy of the excitation source (hν = 40 eV in our work), E_{cutoff} is the energy of secondary electron cutoff, and E_{VBM} is the valence band maxima of samples *vs* NHE at pH = 0.

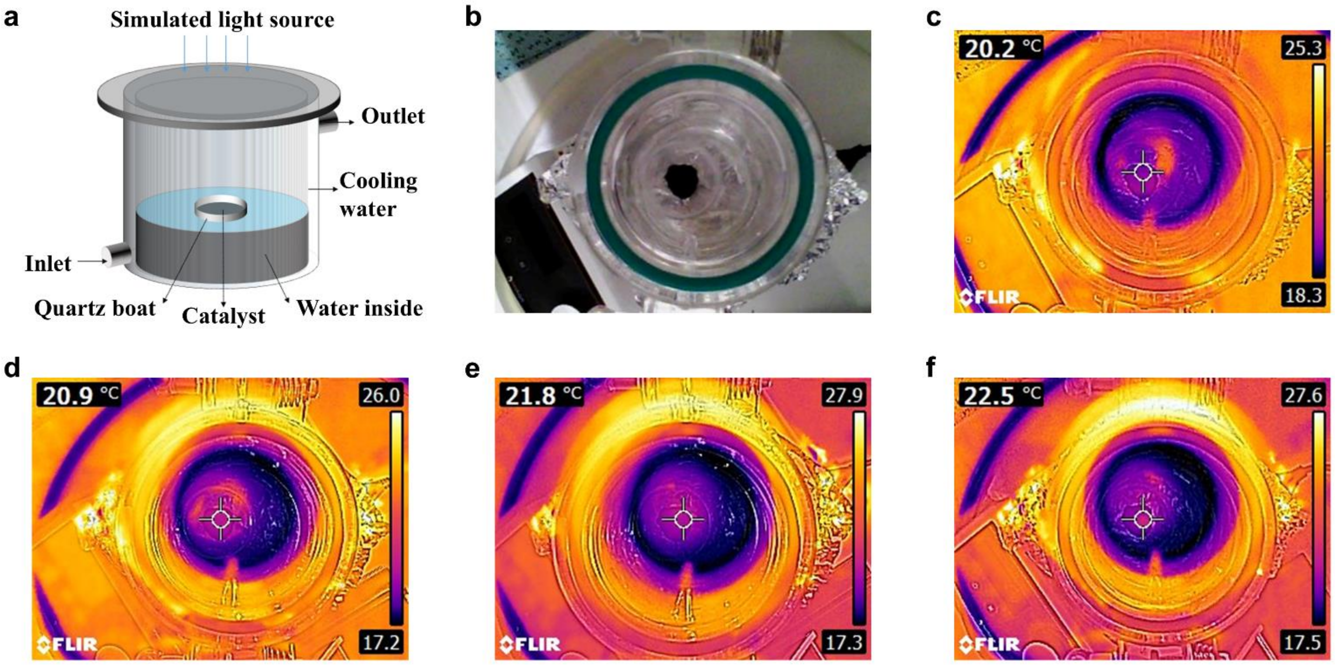

Supplementary Figure 35. (a) Schematic diagram and (b) photograph of the photocatalytic reactor. (c)-(f) *In situ* thermographic photographs during photocatalysis (c: 0h; d: 4h; e: 8h; f: 12h). Thermographic photographs measured by FLIR E8 pro. The dotted white circles indicate the location of the CZTS-S_v nanosheets, and the provided temperatures correspond to the average temperatures of the catalyst thin film during visible light irradiation.

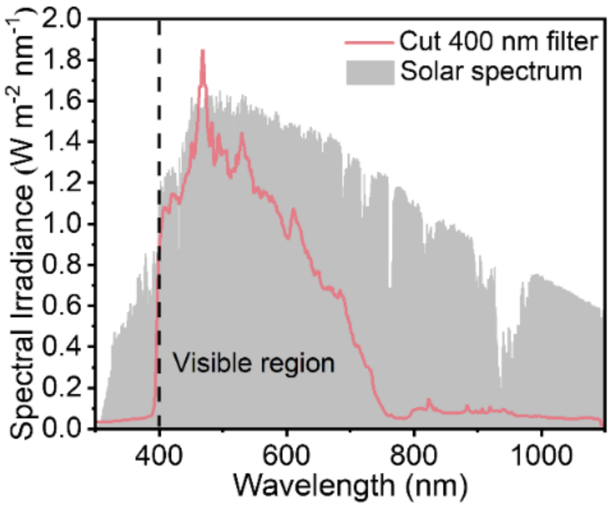

Supplementary Figure 36. The illumination spectrum of our light simulator comparing with sunlight. Cut-400 filter was used to simulate the visible light.

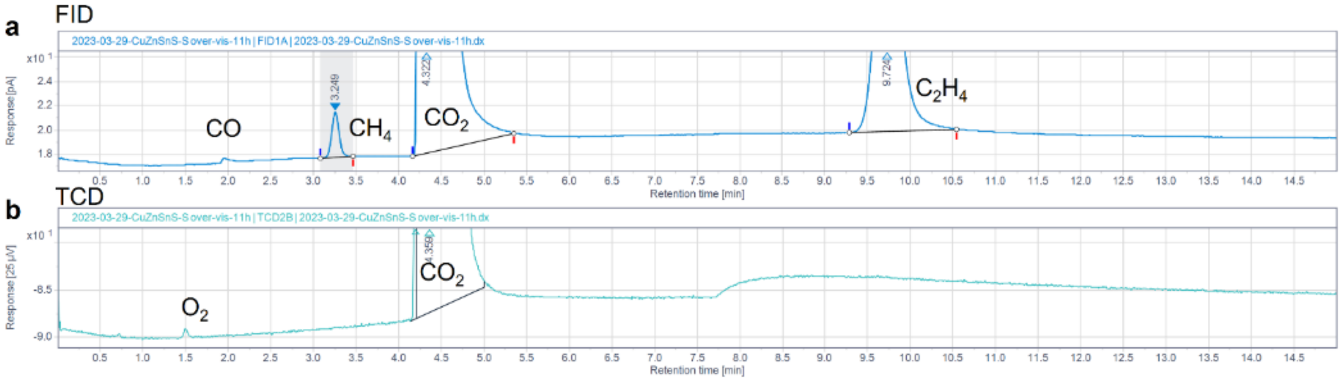

Supplementary Figure 37. Gas products detected by GC after 11h photocatalysis over CZTS-S_v nanosheets. (a) FID signal. (b) TCD signal. The gas products include O_2, CO, CH_4 and C_2H_4.

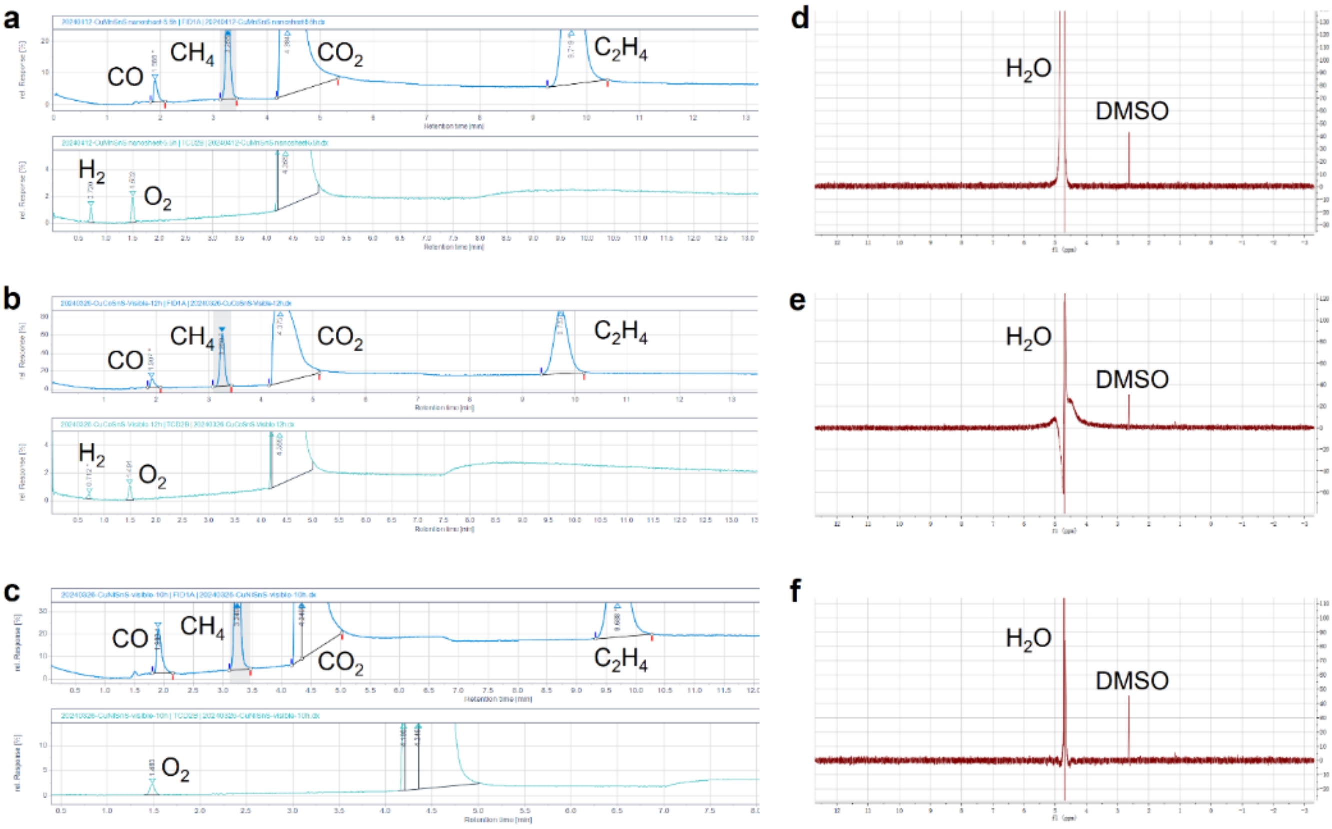

Supplementary Figure 38. The product analysis of multi-site $Cu_2M_iSnS_4$-S_v (M_i=Mn, Co, Ni) nanosheet for photocatalytic CO_2 reduction. GC spectra for (a) CMTS-S_v nanosheet, (b) CCTS-S_v nanosheet and (c) CNTS-S_v nanosheet. ^{1}H NMR spectra for (d) CMTS-S_v nanosheet, (e) CCTS-S_v nanosheet and (f) CNTS-S_v nanosheet.

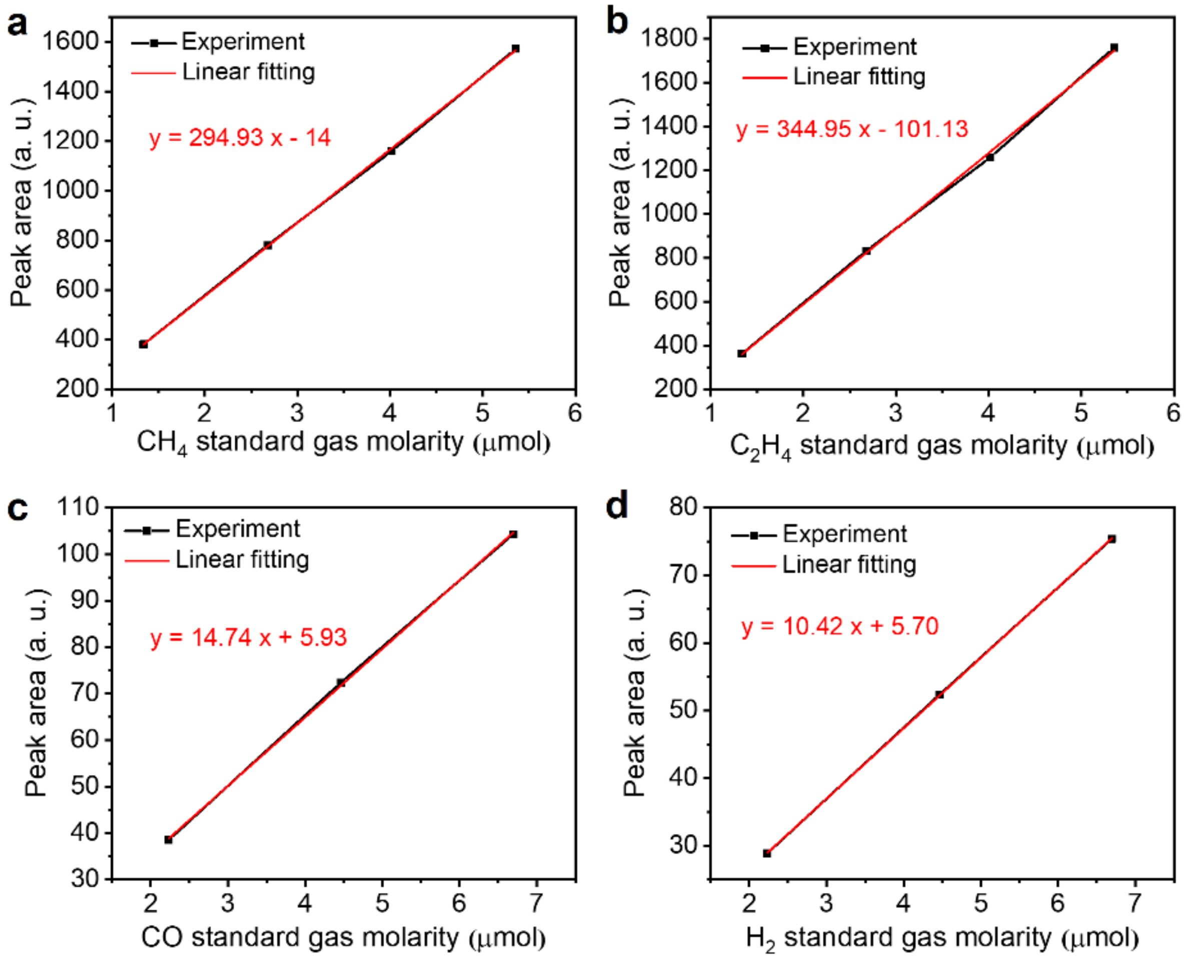

Supplementary Figure 39. The Calibration curves. The standard curves of (a) CH_4, (b) C_2H_4, (c) CO and (d) H_2 for calculating gas products after photocatalysis.

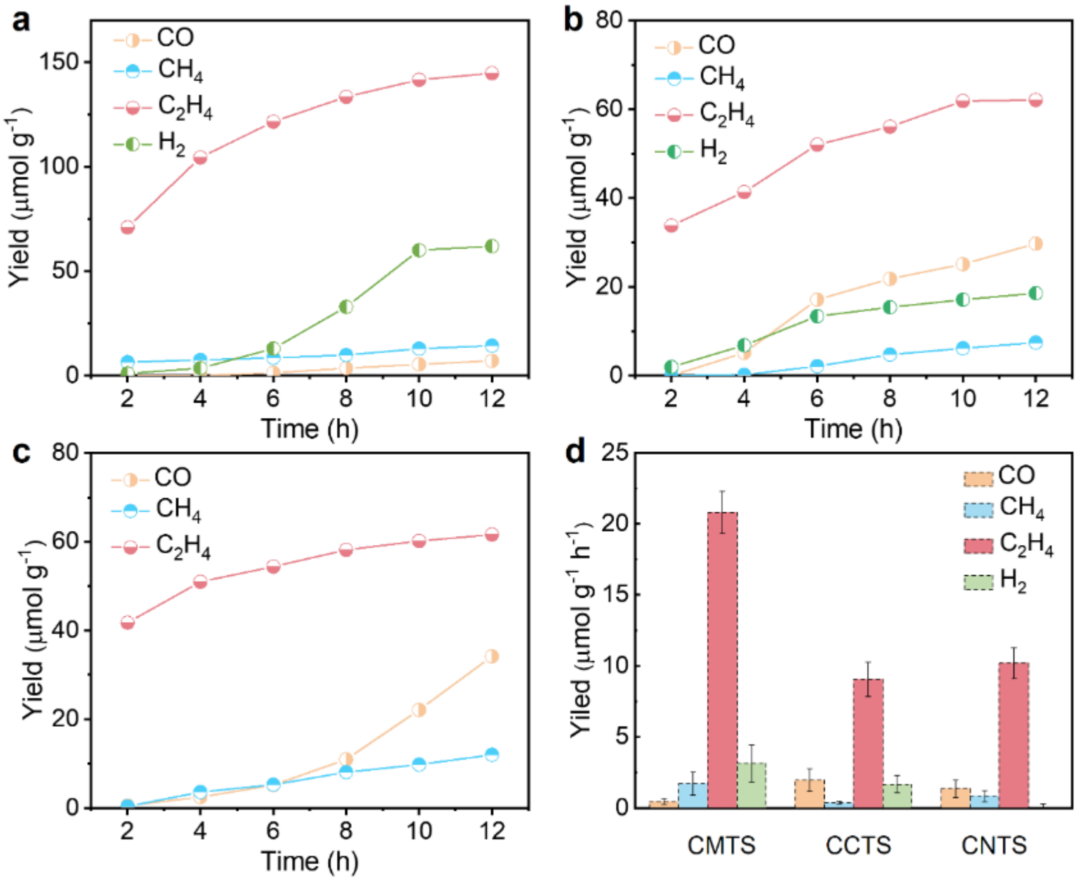

Supplementary Figure 40. The yields of photocatalytic CO_2 reduction to CO, CH_4, C_2H_4 and H_2 using (a) CMTS-S_v, (b) CCTS-S_v and (c) CNTS-S_v nanosheets. (d) Product distribution using different catalysts, in which error bars represent the standard deviation (s. d.) of three independent measurements using fresh samples for each measurement.

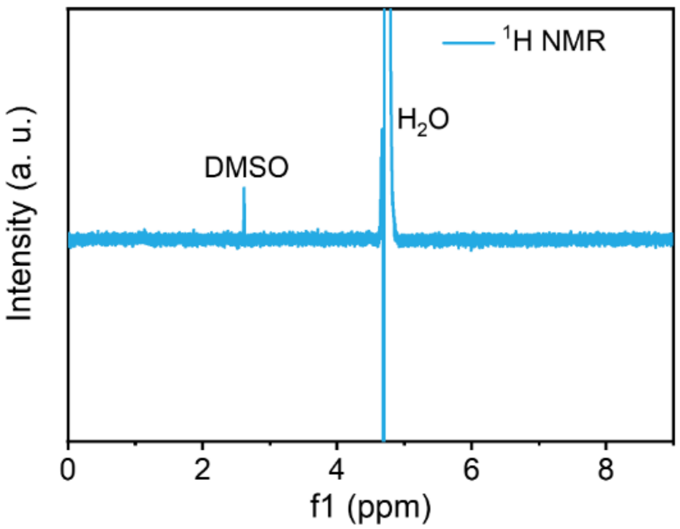

Supplementary Figure 41. Nuclear magnetic resonance (NMR) spectrum. ^1H NMR of CZTS-S_v nanosheet after 12 hours, in which no liquid product is detected.

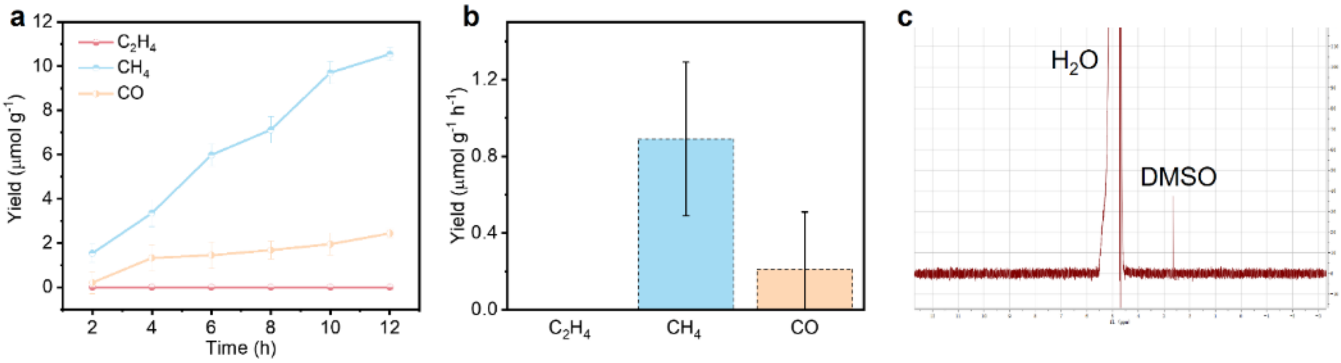

Supplementary Figure 42. Performance of CZTS bulk for visible light-driven CO_2 reduction. (a)-(b) The yields of photocatalytic CO_2 reduction to CO, CH_4 and C_2H_4 using CZTS bulk, in which error bars represent the standard deviation (s. d.) of three independent measurements using fresh sample for each measurement. (c) ^1H nuclear magnetic resonance (NMR) spectrum of CZTS bulk after 12 hours, in which no liquid product is detected.

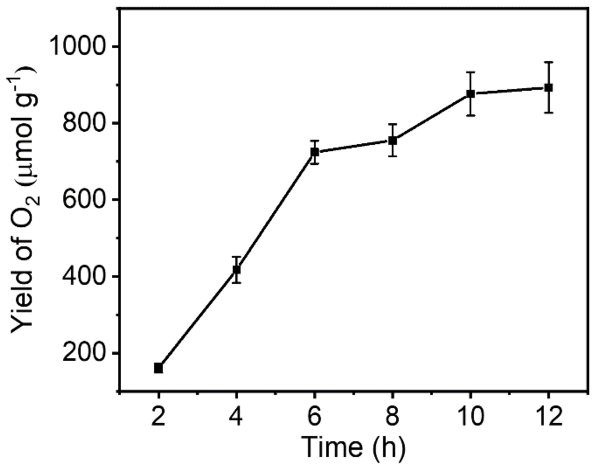

Supplementary Figure 43. The yield of O_2 generation from the water oxidation during the photocatalysis using the CZTS-S_v nanosheets. Error bars represent the standard deviation (s. d.) of three independent measurements using fresh samples for each measurement. Only O_2 gas was detected, the dissolved oxygen and other oxidation products were not taken into account.

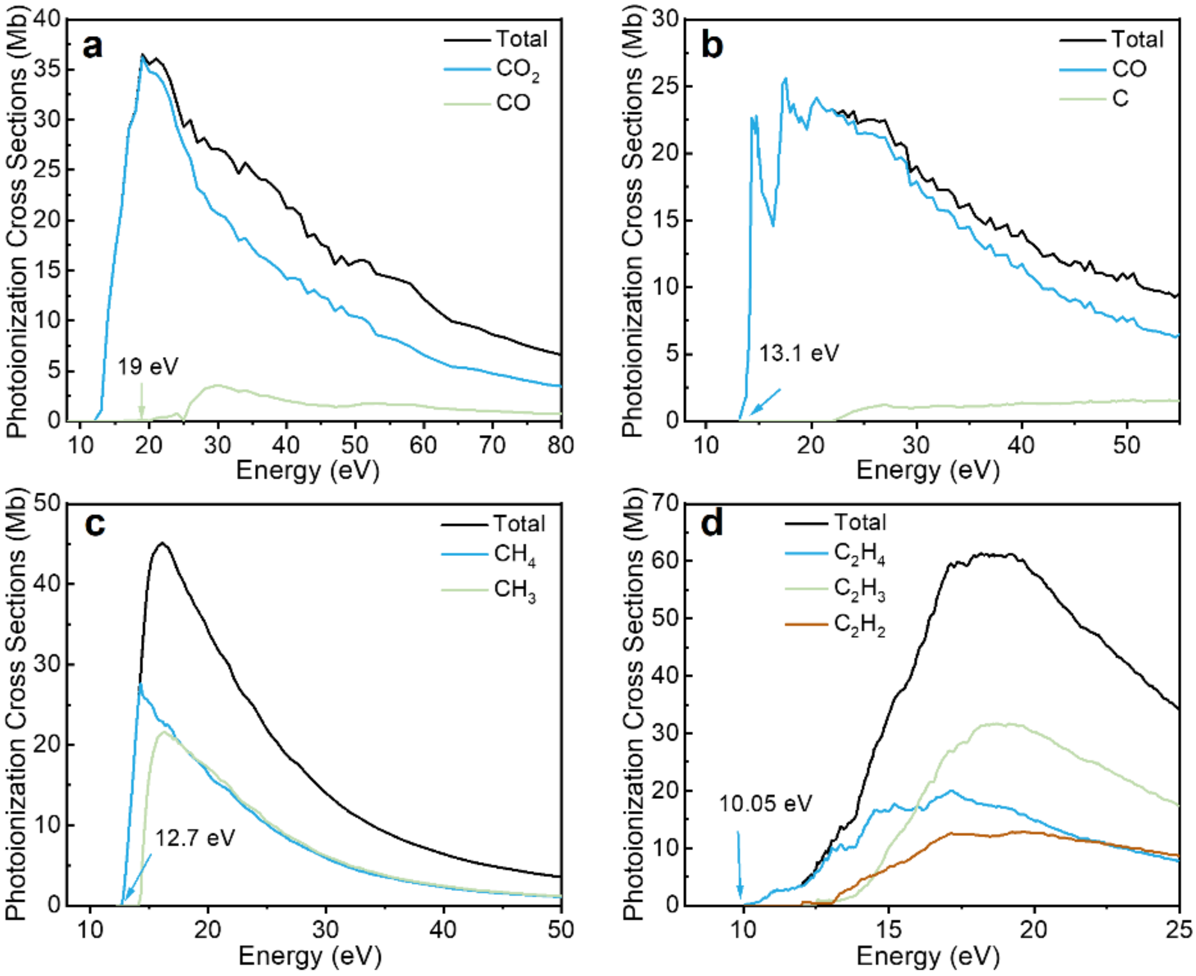

Supplementary Figure 44. Synchrotron-based vacuum ultraviolet photoionization mass spectrometry (SVUV-PIMS). Absolute photoionization cross sections for (a) CO_2, (b) CO, (c) CH_4 and (d) C_2H_4. *http://flame.nsrl.ustc.edu.cn/database/data.php* Supplementary Figure 44a reveals that CO_2 would dissociate into CO when the photon energy approaches about 19 eV; meanwhile, the pure CO and CH_4 can be detected when the photon energy is up to 13.1 eV and 12.7 eV, respectively (Supplementary Figure 44b-c). As such, it is feasible to utilize SVUV-PIMS spectra at the photon energy of 14.5 eV for distinguishing whether CO, CH_4 and C_2H_4 is obtained from CO_2 reduction or dissociation.

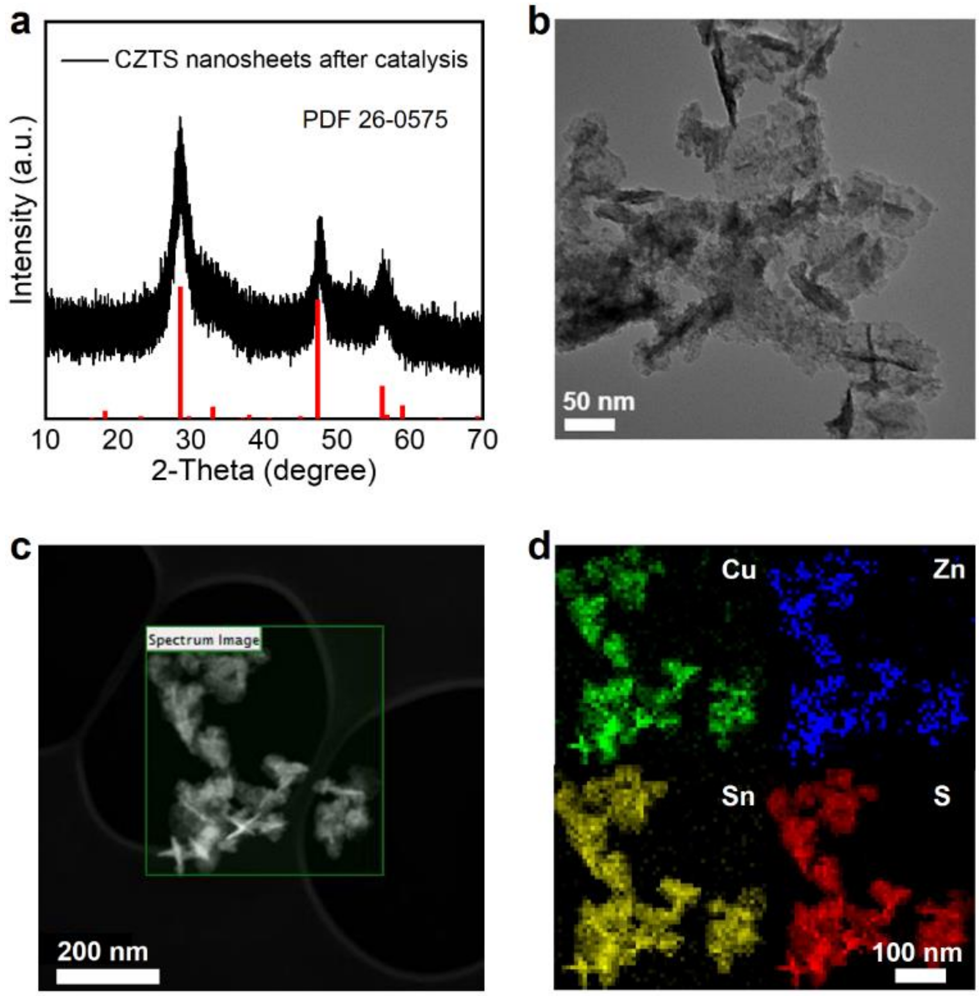

Supplementary Figure 45. Characterizations of CZTS-S_v nanosheet after continuous photocatalysis for 96 h. (a) XRD pattern. (b) TEM image. (c) STEM image. (d) EDS mapping images.

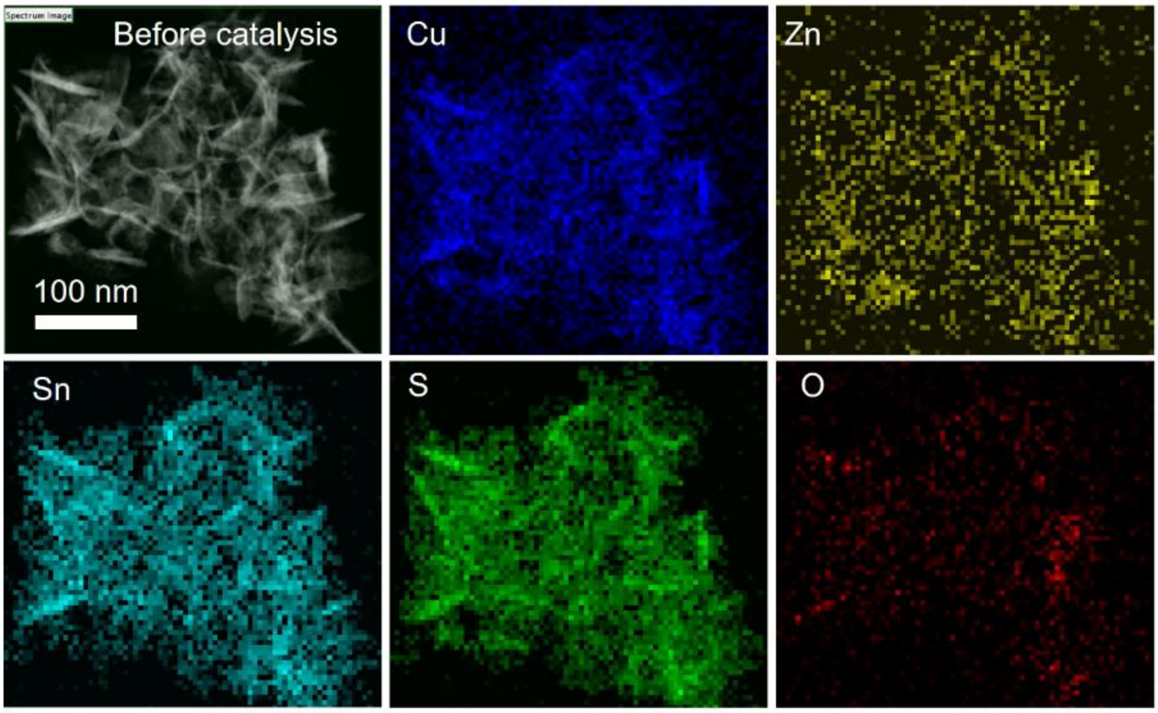

EDS element content of TEM						
	Element	Shell	Signal (Counts)	Comp. (at.%)	k-Factor (/Si)	Det. Corr.
Before catalysis	Cu	K	2080	28.5	1.794	1.00
	Zn	K	1120	15.7	1.900	1.00
	Sn	L	870	11.5	3.195	0.97
	S	K	2510	40.5	1.063	0.93
	**O**	**K**	**100**	**3.8**	**1.251**	**0.79**

Supplementary Figure 46. EDS mapping and content analysis of CZTS-S_v nanosheet before photocatalysis. The content of oxygen element is around 3.8 at.%.

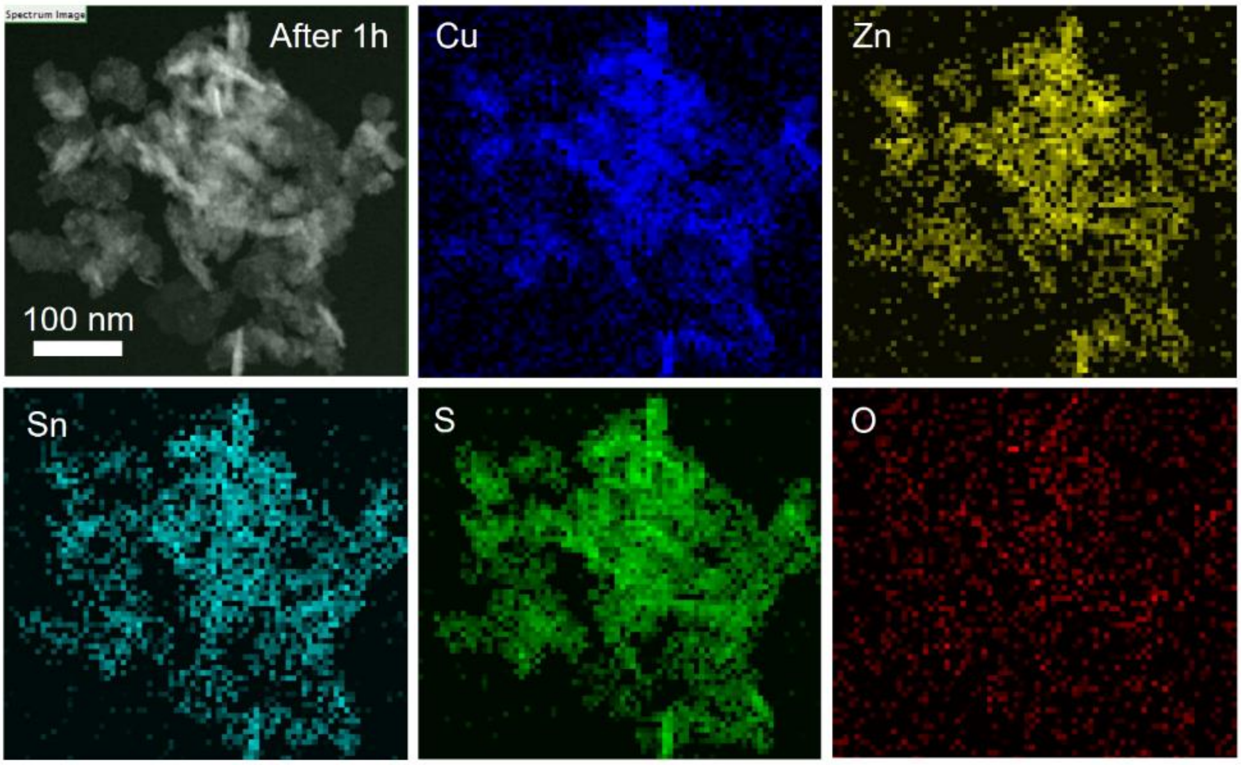

EDS element content of TEM						
	Element	Shell	Signal (Counts)	Comp. (at.%)	k-Factor (/Si)	Det. Corr.
After catalysis for 1 h	Cu	K	3810	34.7	1.794	1.00
	Zn	K	1200	11.3	1.900	1.00
	Sn	L	1410	12.2	3.195	0.97
	S	K	3520	37.7	1.063	0.93
	**O**	**K**	**163**	**4.1**	**1.251**	**0.79**

Supplementary Figure 47. EDS mapping and content analysis of CZTS-S_v nanosheet after photocatalysis for 1h. The content of oxygen element is around 4.1 at.%.

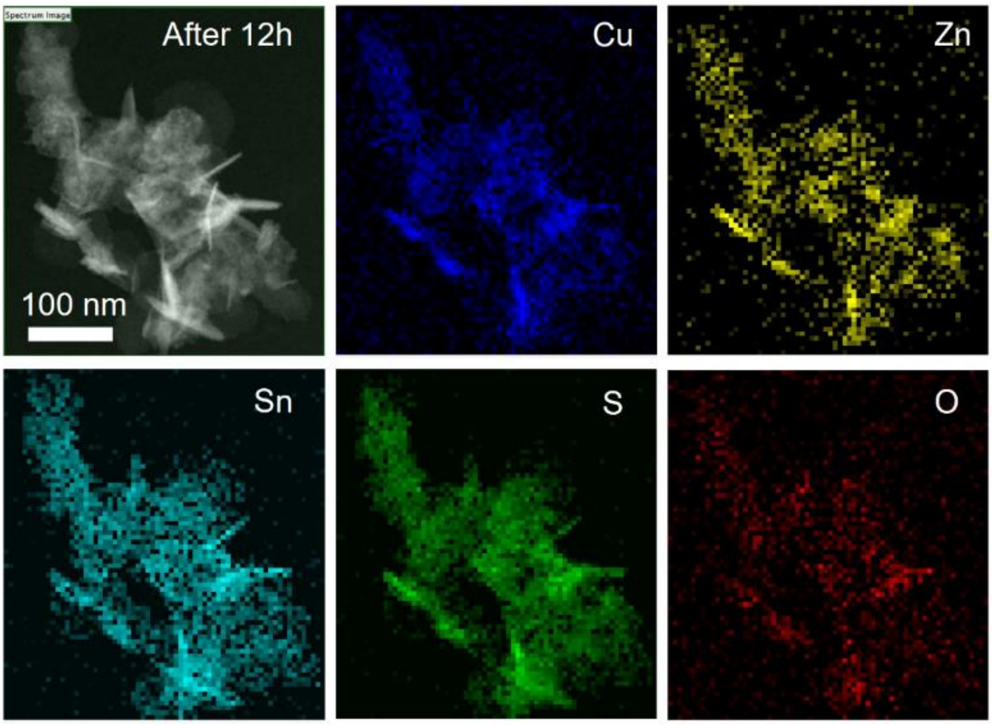

EDS element content of TEM						
	Element	Shell	Signal (Counts)	Comp. (at.%)	k-Factor (/Si)	Det. Corr.
After catalysis for 12 h	Cu	K	8670	30.3	1.794	1.00
	Zn	K	2970	10.7	1.900	1.00
	Sn	L	5070	16.9	3.195	0.97
	S	K	9290	38.1	1.063	0.93
	**O**	**K**	**414**	**4.0**	**1.251**	**0.79**

Supplementary Figure 48. EDS mapping and content analysis of CZTS-S_V nanosheet after photocatalysis for 12h. The content of oxygen element is around 4.0 at.%.

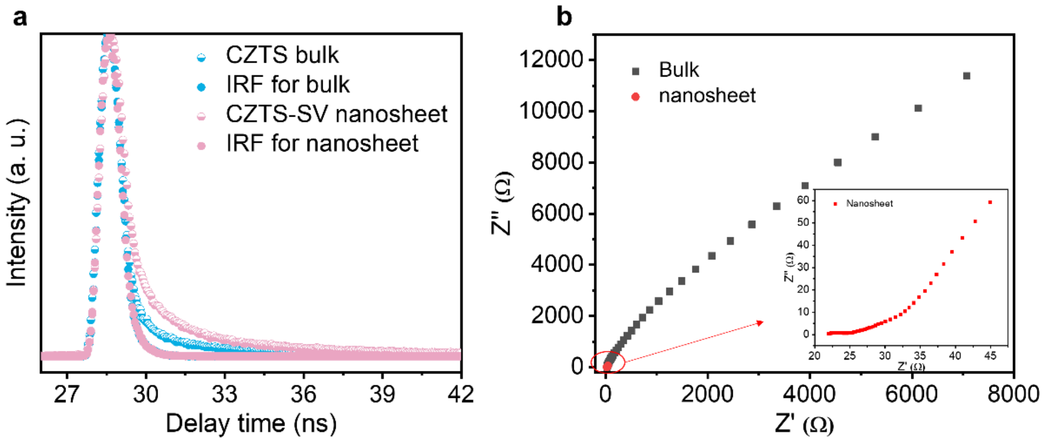

Supplementary Figure 49. Charge carrier dynamics studies. (a) The time-resolved fluorescence spectra with instrument response function (IRF) and (b) Nyquist plots of electrochemical impedance spectroscopy (EIS) of CZTS bulk and nanosheet.

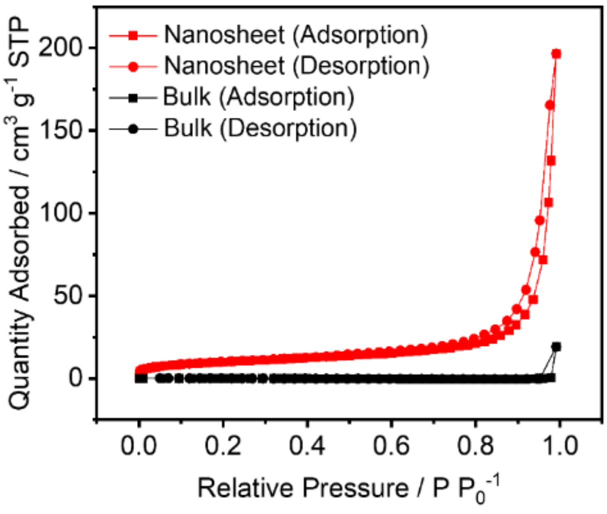

Supplementary Figure 50. BET isotherms for CZTS bulk and nanosheet. The BET surface area for CZTS-S_v nanosheet is 36.04 m^2 g^{-1}, which is more than 36 times than that of CZTS bulk (<1.00 m^2 g^{-1}), fairly agreeing with the corresponding CO_2 adsorption isotherms.

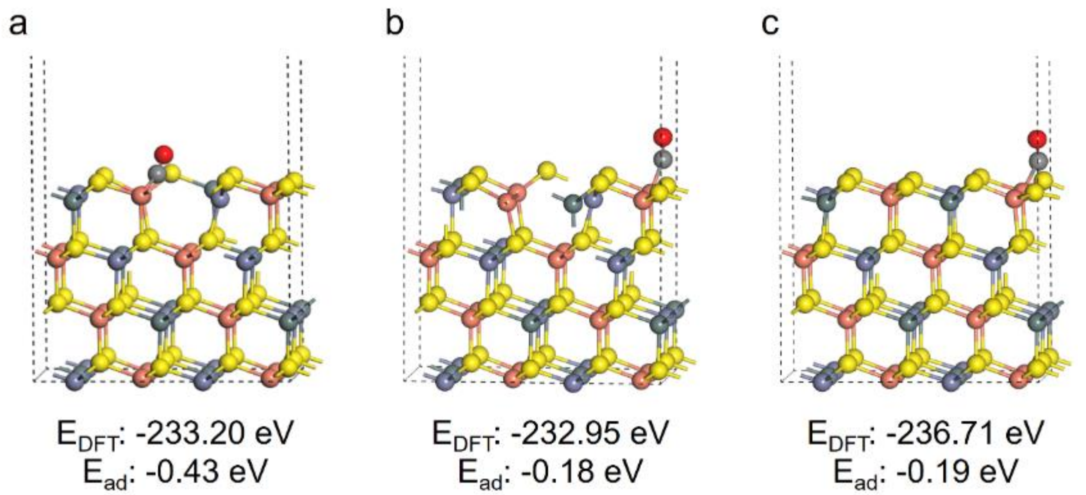

Supplementary Figure 51. The calculated total energy (E_{DFT}) and adsorption energy (E_{ad}) of CO* species at different Cu sites. (a) The low-coordination Cu site in CZTS-S_v slab. (b) The saturated Cu site in CZTS-S_v slab. (c) The saturated Cu site in pristine CZTS slab. The adsorption energy of CO* at the low-coordination Cu site in CZTS-S_v slab (-0.43 eV) is stronger than that at the saturated Cu site in both CZTS-S_v (-0.18 eV) and pristine CZTS (-0.19 eV) slab.

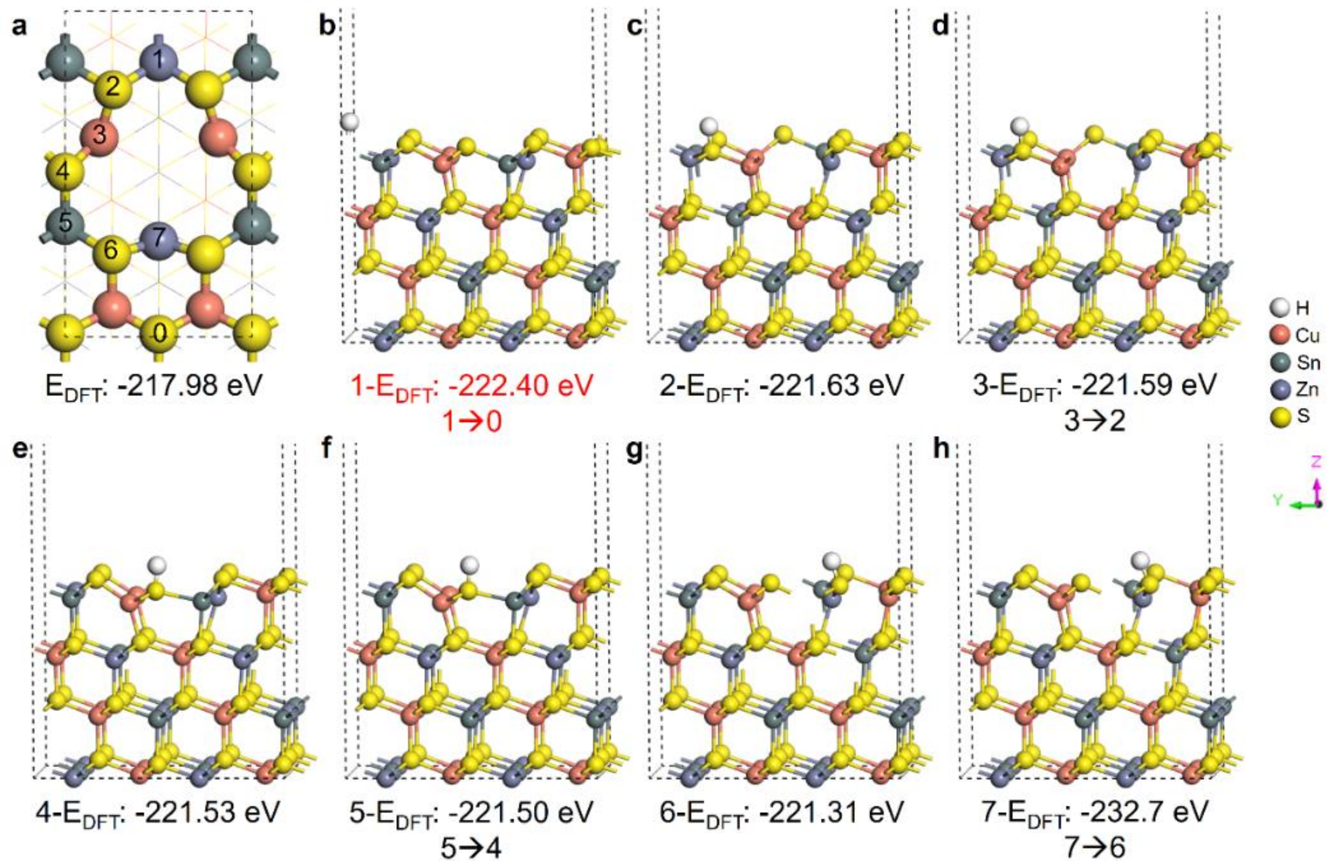

Supplementary Figure 52. The theoretical models of H adsorption on the CZTS-S_v slab. (a) The possible adsorption sites (top view). (b)-(h) The H adsorption models and calculated total energy (E_{DFT}) for H adsorbed at 1-7 sites (0 site is the S site adjacent to 1 site). Notably, the H atoms adsorbed on 1, 3, 5 sites prefer to transfer to the adjacent S sites (0, 2, 4), respectively, indicating that the surface S atoms can act as the proton accepter to provide more H* for CO_2 reduction. The first (b) model is considered as the most stable adsorption structure with H adsorption energy of -1.04 eV.

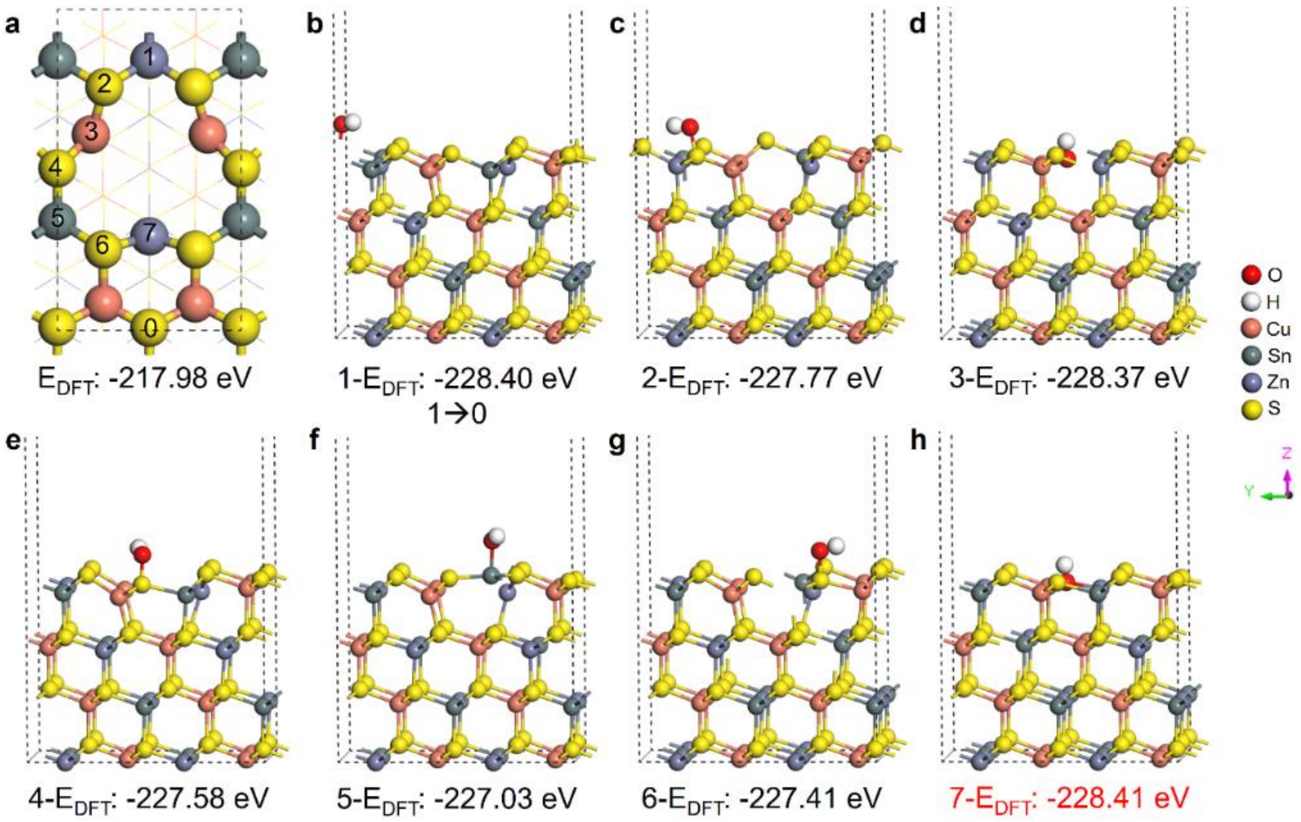

Supplementary Figure 53. The theoretical models of OH adsorption on the CZTS-S_v slab. (a) The possible adsorption sites (top view). (b)-(h) The OH adsorption models and calculated total energy (E_{DFT}) for OH adsorbed at 1-7 sites (0 site is the S site adjacent to 1 site). Notably, the OH atoms adsorbed on 1 sites prefer to transfer to the adjacent S sites (0 site). The seventh (h) model is considered as the most stable adsorption structure with OH adsorption energy of -2.71 eV.

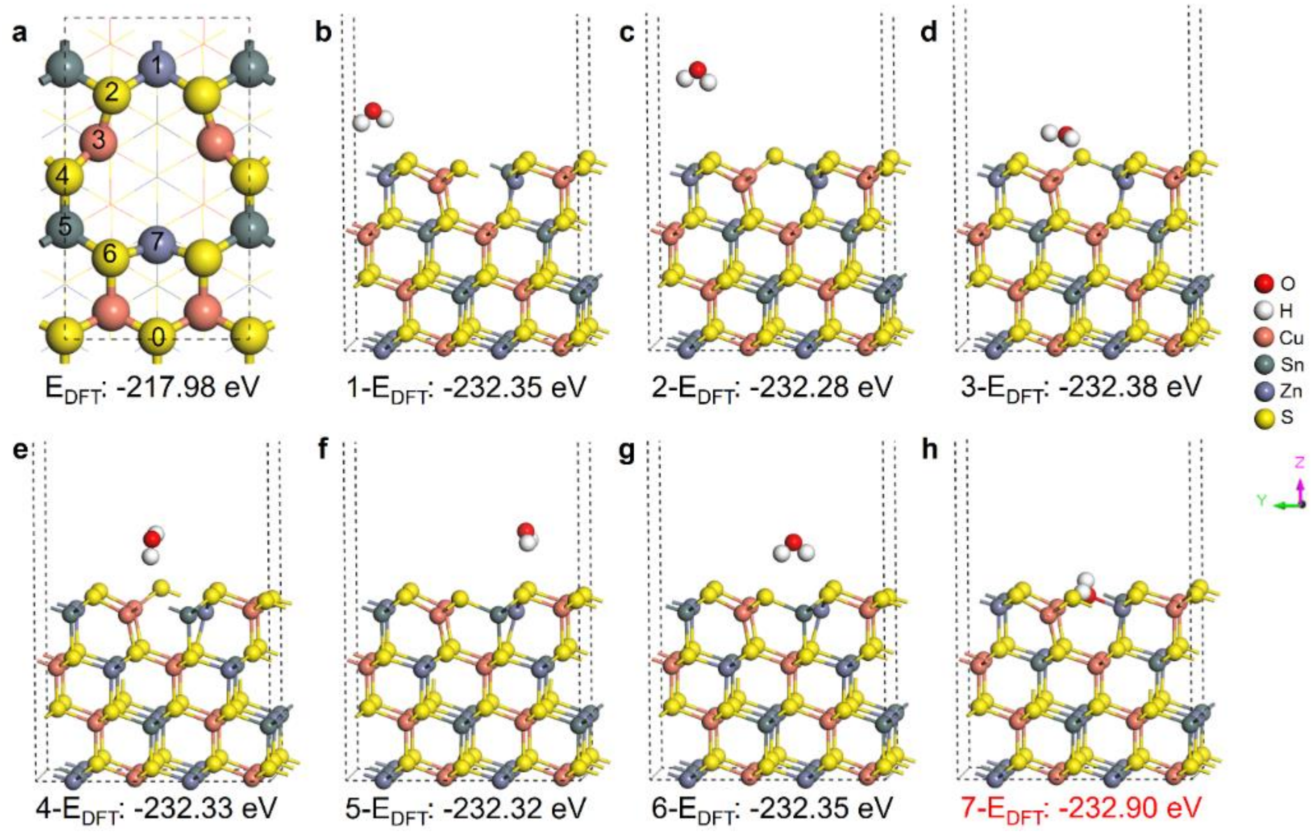

Supplementary Figure 54. The theoretical models of H_2O adsorption on the CZTS-S_v slab. (a) The possible adsorption sites (top view). (b)-(h) The H_2O adsorption models and calculated total energy (E_{DFT}) for H_2O adsorbed at 1-7 sites (0 site is the S site adjacent to 1 site). The seventh (h) model is considered as the most stable adsorption structure with H_2O adsorption energy of -0.71 eV.

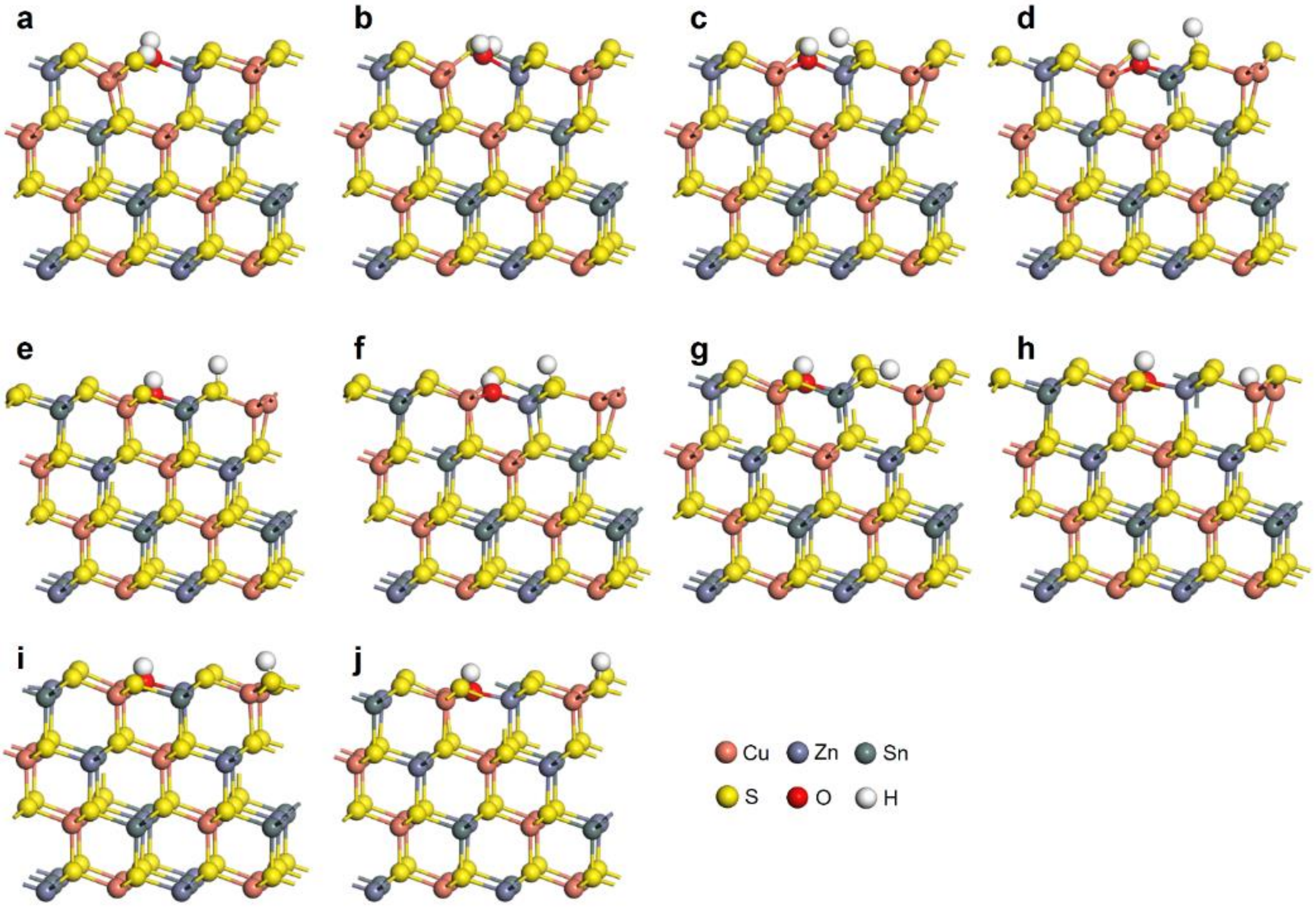

Supplementary Figure 55. TS of H_2O splitting processes to H* and OH* intermediates on the surface of the CZTS-S_v slab. (a)-(j) The TS models. The TS are calculated by the CI-NEB method, in which the energy barrier is computed to 1.38 eV.

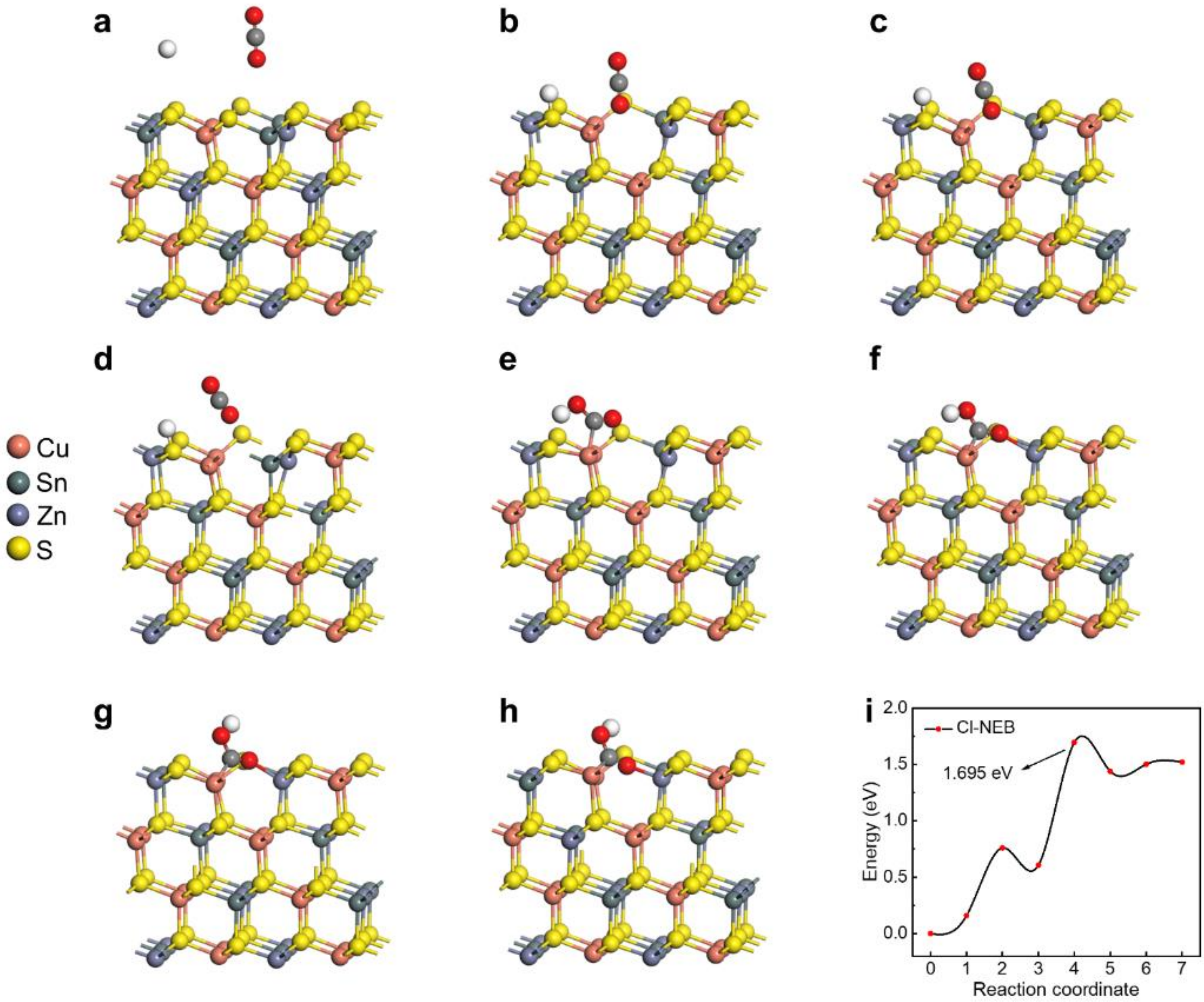

Supplementary Figure 56. TS of CO_2 → COOH* processes on the surface of the CNTS-S_v slab. (a)-(h) The TS models. (i) The calculated TS energy plots. The TS are calculated by the CI-NEB method, in which the energy barrier is computed to 1.695 eV.

Supplementary Tables

Supplementary Table 1. Total energy and free energy correction for species and reaction intermediates over the CZTS-S_V nanosheet (*) during CO_2 reduction to C_2H_4.

Unit (eV)	E_{DFT}	ZPE	TΔS
H_2	-6.76	0.27	0.40
CO_2	-22.98	0.31	0.66
H_2O	-14.21	0.56	0.67
CO	-14.79	0.13	0.6
C_2H_4	-31.97	1.35	0.68
*	-217.98	\	\
COOH*	-243.60	0.60	0.23
CO*	-233.20	0.18	0.18
CO* + COOH*	-259.26	0.82	0.42
CO* + CO*	-247.72	0.37	0.27
CO* + COH*	-250.51	0.71	0.25
COCOH*	-250.02	0.70	0.26
COC*	-240.13	0.34	0.18
COCH*	-244.35	0.59	0.22
$COCH_2$*	-248.97	0.88	0.28
$CHOCH_2$*	-252.96	1.23	0.19
O*	-223.12	0.07	0.06

Supplementary Table 2. Gibbs free energy (eV) of CO_2 reduction to C_2H_4 over the CZTS-S_v nanosheets.

ΔG (* + CO_2 + H_2O)	ΔG (COOH * + CO_2)	ΔG (CO* + H_2O)	ΔG (CO* + COOH*)	ΔG (CO* + CO*)	ΔG (COH* + CO*)	ΔG (COCOH*)	ΔG (COC*)	ΔG (COCH*)	ΔG ($COCH_2$*)	ΔG ($COHCH_2$*)	ΔG (O* + C_2H_4)
0	1.52	0.68	1.80	2.16	3.18	3.65	2.38	1.82	0.87	0.77	1.72

Supplementary References

1. Scanlon, D.O. et al. Understanding conductivity anomalies in CuI-based delafossite transparent conducting oxides: Theoretical insights. *J. Chem. Phys.* **132**, 024707 (2010).
2. Zasada, F. et al. Surface structure and morphology of M[CoM']O_4 (M = Mg, Zn, Fe, Co and M' = Ni, Al, Mn, Co) spinel nanocrystals-DFT+U and TEM screening investigations. *J. Phys. Chem.* **118**, 19085-19097 (2014).
3. Aykol, M. et al. Local environment dependent GGA+U method for accurate thermochemistry of transition metal compounds. *Phys. Rev. B* **90**, 115105 (2014).
4. Kang, J. et al. Valence oscillation and dynamic active sites in monolayer NiCo hydroxides for water oxidation. *Nat. Catal.* **4**, 1050-1058 (2021).
5. Tkalych, A. J. et al. A density functional +U assessment of oxygen evolution reaction mechanisms on β-NiOOH. *ACS Catal.* **7**, 5329-5339 (2017).
6. Schleife, A. et al. Optical and energy-loss spectra of MgO, ZnO, and CdO from ab initio many-body calculations. *Phys. Rev. B* **80**, 035112 (2009).
7. Singh, A.K. et al. Phase transitions of bipartite entanglement. *Phys. Rev. Lett.* **101**, 055502 (2008).
8. Tseberlidis, G. et al. Band-gap tuning induced by germanium introduction in solution-processed kesterite thin films. *ACS Omega* **7**, 23445-23456 (2022).
9. Kattan, N. et al. Crystal structure and defects visualization of Cu_2ZnSnS_4 nanoparticles employing transmission electron microscopy and electron diffraction. *Appl. Mater. Today* **1**, 52-59 (2015).
10. Guo, Q. et al. Synthesis of Cu_2ZnSnS_4 nanocrystal ink and its use for solar cells. *J. Am. Chem. Soc.* **131**, 11672-11673 (2009).
11. Peng, H. et al. Defective $ZnIn_2S_4$ nanosheets for visible-light and sacrificial-agent-free H_2O_2 photosynthesis via O_2/H_2O redox. *J. Am. Chem. Soc.*(2023). DOI: 10.1021/jacs.3c10390
12. Luo, N. et al. S defect-rich ultrathin 2D MoS_2: The role of S point-defects and S stripping-defects in the removal of Cr(VI) via synergistic adsorption and photocatalysis. *Appl. Catal. B: Environ*. **299**, 120664 (2021).
13. Wang, W. et al. Photocatalytic C-C coupling from carbon dioxide reduction on copper oxide with mixed-valence copper(I)/copper(II). *J. Am. Chem. Soc.* **143**, 2984-2993 (2021).

14. Zhu, S. et al. Selective CO_2 photoreduction into C_2 product enabled by charge-polarized metal pair sites. *Nano Lett* **21**, 2324-2331 (2021).
15. Wang, L. et al. Black indium oxide a photothermal CO_2 hydrogenation catalyst. *Nat. Commun.* **11**, 2432 (2020).
16. Yan, T. et al. Bismuth atom tailoring of indium oxide surface frustrated Lewis pairs boosts heterogeneous CO_2 photocatalytic hydrogenation. *Nat. Commun.* **11**, 6095 (2020).
17. Liu, F. et al. Direct Z-scheme hetero-phase junction of black/red phosphorus for photocatalytic water splitting. *Angew. Chem. Int. Ed.* **58**, 11791-11795 (2019).
18. Zhao, D. et al. Boron-doped nitrogen-deficient carbon nitride-based Z-scheme heterostructures for photocatalytic overall water splitting. *Nat. Energy* **6**, 388-397 (2021).
