# Supplementary material for: Designing multi-metal-site nanosheet catalysts for CO2 photoreduction to ethylene
Source: Nat Commun. 2025 Jul 15;16:6500. doi: 10.1038/s41467-025-61850-7 (PMC12259931; doi:10.1038/s41467-025-61850-7)
Supplement: Supplementary file 2 — Description of Additional Supplementary Files [file 41467_2025_61850_MOESM2_ESM.pdf]

Description of Additional Supplementary Files

File Name: Supplementary Data 1
Description: Theoretical models for DFT calculations
